# Long-read, multi-amplicon sequencing to explore genetic diversity associated with starch degrading phenotypes in amylolytic *Lactobacillaceae*

**DOI:** 10.3389/fmicb.2025.1548052

**Published:** 2025-03-26

**Authors:** Sandra A. Olivier, Michelle K. Bull, John P. Bowman, Tom Ross, Belinda Chapman

**Affiliations:** ^1^Agriculture and Food Systems, Tasmanian Institute of Agriculture University of Tasmania, Hobart, TAS, Australia; ^2^Quantal Bioscience, Sydney, NSW, Australia

**Keywords:** *Lactobacillaceae*, amplicon sequencing, nanopore, gene assay, phenotype, amylolytic, starch

## Abstract

Characterizing starch-degrading *Lactobacillaceae* and associated enzymes remains relevant as various industries seek to harness their activity to produce valuable by-products, develop novel food applications, and to aid the sustainable bioconversion of starch-rich resources. To support this, we developed a targeted methodological and analysis framework utilizing complimentary phenomic and genomic assays informative of the starch degrading potential of *Lactobacillaceae*. Adapted starch agar plate assays incorporating diversified starch sources and states facilitated the rating of extracellular amylolytic activity by starch-processing-line isolates [*Lactobacillus amylovorus* (*n* = 3), *Lactobacillus amylolyticus* (*n* = 2), and *Limosilactobacillus reuteri* (*n* = 2)] as weak to moderate based on the complete or partial hydrolysis of retrograded soluble (SS), or potato and wheat (WS), starches, respectively, and the partial hydrolysis of raw SS. In contrast, the known raw starch degrader, *L. amylovorus* NRRL B4540, was rated as strong, with complete hydrolysis of all retrograded starch sources and raw WS. To explore genetic diversity and the putative enzymes associated with phenotypic diversity amongst *L. amylovorus* and *L*. *amylolyticus*, a multi-amplicon sequencing approach using MinION™ was used to simultaneously sequence starch-degradation-associated genes identified from them. Gene and deduced amino acid sequence analysis suggested raw starch hydrolysis by *L. amylovorus* NRRL B4540 was largely attributed to a*myA* encoding a rare α-amylase with unique starch binding domain (targeting α-1,4 linkages), but which was predicted to also require the starch debranching activity (targeting α-1,6 linkages) associated with (putative) *pul*-encoded pullulanase (Pul) for complete hydrolysis. Without *amyA*, Pul was hypothesized necessary for observed starch degradation by *L. amylovorus* and *L. amylolyticus* test isolates; as a previously undescribed amylopullulanase with dual activity, or as a pullulanase requiring complimentary α-1,4 activity from an additional enzyme, potentially Gly2 (a putative maltogenic α-amylase). Whilst further work is required to characterize these enzymes, including those encoded by gene variants, the experimental approach described here provided the necessary evidence to warrant this. Further, this framework is likely adaptable for the direct analysis of *Lactobacillaceae*-rich microbiomes for amylolytic potential and for the targeted screening of various other functions across different taxa.

## 1 Introduction

The family *Lactobacillaceae* represents the largest and most diverse group of lactic acid bacteria (LAB) (Zheng et al., 2020). Due to their significant metabolic versatility, LAB are recognized for their important role in food manufacturing, human and animal health, and various industrial applications (Reddy et al., 2008; Kerketta et al., 2023). Starch degradation by LAB is a metabolic trait of particular interest given their direct conversion of starch into monosaccharides as well as lactic acid (Velikova et al., 2016). A review of reported amylolytic LAB (ALAB) recognized 28 species from a diverse range of *Lactobacillaceae* genera. These species included *Lactobacillus amylovorus, Lactobacillus amylolyticus* and the genus, *Amylolactobacillus* (Petrova et al., 2022). These taxa were found to possess unique genetic adaptations that enable efficient starch breakdown (Petrova et al., 2013, 2022; Velikova et al., 2016). However, outside of these innately amylolytic species, amylolytic activity is usually strain-specific (Petrova and Petrov, 2012). Amylolytic LAB have been isolated from a variety of starch-rich environments where they dominate, including agricultural settings (silage), fermented foods and the gastrointestinal tract of animals and humans (Reddy et al., 2008; Petrova et al., 2013, 2022).

Starch, a polysaccharide composed of two α-glucans, amylose and amylopectin, is an abundant resource used in numerous food production and industrial applications (Bart et al., 2013; Petrova et al., 2013, 2022). Notably, starch is used as a fermentable substrate to produce a variety of metabolic by-products, while starch-rich foods and silage may be fermented for nutritional enhancement and preservation (Agati et al., 1998; Velikova et al., 2016). Enzymes that hydrolyze starch are classed as glycoside hydrolases (GH) and are largely identified within the GH13 family (Petrova et al., 2013; Janeček and Svensson, 2022) as described in the Carbohydrate-Active enZymes database (CAZy; http://www.cazy.org/) (Drula et al., 2022). Amylase-type enzymes act by cleaving the α-1,4 and/or α-1,6 glycosidic bonds within starch polymers, variably producing oligosaccharides and/or glucose depending on their mode of action (Hii et al., 2012; Roy et al., 2021). Whilst a number of GH13 enzymes with generic hydrolytic activity are produced by microbes, the discovery of those that produce raw starch degrading enzymes is an area of broad interest given the efficiencies they offer in replacing conventional processes designed to break down starch prior to fermentation (Goyal et al., 2005; Reddy et al., 2008; Sun et al., 2010; Moradi et al., 2014). Raw starch degrading enzymes that directly hydrolyze raw (native) starch granules below the starch gelatinization temperature have primarily been identified from the GH13 family and include α-amylases (EC 3.2.1.1), maltogenic α-amylases (EC 3.2.1.133) and amylopullulanases (EC 3.2.1.41) (Sun et al., 2010; Božić et al., 2017; Roy et al., 2021). A feature typical amongst raw starch degrading enzymes is the presence of a domain adjacent to the catalytic domain of the protein, whose function is ascribed to carbohydrate binding, enhancing interaction with the active site of the protein (Rodríguez-Sanoja et al., 2005; Božić et al., 2017; Roy et al., 2021). These are classified as carbohydrate binding molecules (CBM) and vary in their substrate specificity and functional features according to their CAZy database family; CBMs that interact with starch are sometimes referred to as the starch binding domain (SBD) of the protein (Rodríguez-Sanoja et al., 2005).

The distribution of GH13 enzyme-encoding genes across *Lactobacillaceae* is wide (Sun et al., 2015), and the gene sequences for each enzyme, highly variable (Turpin et al., 2011; Humblot et al., 2014). This implies an inherent difficulty in screening *Lactobacillaceae* for enzyme-specific starch degrading genes using universal primers, and points to the use of whole genome sequencing (WGS) to facilitate the identification of genes of interest. However, the sequencing depth, bioinformatic expertise and cost of a WGS approach can be prohibitive, and unnecessarily exhaustive. Instead, the development of strategic, targeted amplicon sequencing of starch degrading genes relevant to particular species and/or substrate pathways could be implemented for screening purposes. Conceptually, this has been successfully demonstrated in previous studies exploring starch degrading gene composition (Turpin et al., 2011) and expression (Humblot et al., 2014; Velikova et al., 2016) in ALAB. Beyond gene detection, however, there is informative value in examining gene sequences; whether to monitor for gene sequence variants or to explore the predicted conserved domains of deduced protein sequences for functional clues (Petrova and Petrov, 2012). Given the average gene is ~900 bp in prokaryotes (Xu et al., 2006), Oxford Nanopore Technologies' (ONT; Oxford, United Kingdom) long-read sequencing platform with Q20+ chemistry (>99% read accuracy) can be implemented to sequence and analyze whole genes with confidence (Oxford Nanopore Technologies, 2023; Zhang T. et al., 2023).

Because the detection of genes encoding starch degrading enzymes is not always indicative of expressed activity (Turpin et al., 2011; Petrova et al., 2013; Stefanovic and McAuliffe, 2018), forgoing the complexities of transcriptomic analysis, the implementation of simplified, yet informative, phenotypic assays that complement targeted genomic screening can provide the evidence necessary to warrant further investigation of ALAB of interest. “Gold standard” starch agar plate assays measure zones of clearing in (previously gelatinized) soluble starch as a diagnostic feature of ALAB that produce extracellular enzymes (American Society for Microbiology, 2012; Petrova et al., 2013). This is significant because the production of extracellular starch degrading enzymes is associated with a better conversion rate of starch and is therefore a phenotype that is sought as an indicator of functionally important ALAB (Petrova and Petrov, 2012; Petrova et al., 2013; Moradi et al., 2014; Velikova et al., 2016). To identify ALAB with stronger and/or more diverse hydrolytic potential, including those capable of raw starch degradation (RSD), starch agar plate protocols can be adapted to include different starch sources and states (e.g., raw).

This study aimed to develop a targeted and efficient methodological and analysis framework implementing complimentary phenomic and genomic assays to characterize the starch-degrading potential of *Lactobacillaceae*, specifically with respect to GH13 enzyme-encoding genes. LAB isolated from a starch processing line were initially screened using adapted starch agar plate protocols designed to identify ALAB with diverse extracellular amylolytic capacity. Selected GH13 enzyme-encoding genes, known for their association with starch degradation in ALAB, were used to develop a PCR-based assay targeting these genes in the ALAB isolates. Using ONT's MinION™ long-read sequencing platform and a multi-amplicon sequencing approach, we simultaneously sequenced multiple genes from various isolates. Subsequent bioinformatic analyses facilitated nucleotide and deduced amino acid sequence comparisons, including comparisons to published strains. The relationship between starch degradation-associated phenotypes and identified genes was explored, considering the predicted translation and putative function of gene-encoded enzymes, particularly in cases where sequence variants were identified. This first-time application of multi-amplicon Nanopore sequencing, combined with complementary phenotyping to profile starch degradation by *Lactobacillaceae*, serves as a use-case demonstrating the utility of targeted screening for deriving rapid functional insights. Such an approach supports efforts to understand the amylolytic potential of LAB-rich communities and potentially identify strains of industrial utility.

## 2 Materials and methods

### 2.1 Isolation of LAB from starch processing line

Samples (~50 mL) were taken from outlet points along a starch processing line and LAB were recovered from these on Lactobacilli MRS agar (MRSA; BD Difco, United States) incubated anaerobically (Anaerogen, Oxoid, United Kingdom) at 15 and 45°C for 10 and 3 d, respectively. Different colony morphotypes were selected and sub-cultured for storage in glycerol (20% v/v) at −80°C.

### 2.2 Phenotypic assessment of starch degradation by LAB

Starch degradation was assessed using a modified MRSA with 1% w/w starch (mMRSA+1%S), based on the modified MRS medium developed for lactobacilli fermentation studies (de Man et al., 1960); 1.5% w/w bacteriological agar (Oxoid, United Kingdom) was added to the basal medium and the optional chlorophenol red indicator was omitted. The carbohydrate sources used were potato starch (PS; 03967-500G; Sigma-Aldrich), wheat starch (WS; S5127-500G; Sigma-Aldrich) or soluble starch (SS; AJA526-500G; Univar, Ajax Finechem, Australia.); the SS comprised an unspecified combination of potato and wheat starch. The mMRSA+1%S was prepared in three different ways to vary the physical state of the starch; (1) A-Retrograded: starch added before autoclaving (121°C/15 min) with plates stored at 25°C for 1 d; (2) B-Retrograded: starch added before autoclaving and plates stored at 4°C for 3 d; and (3) Raw: starch added after autoclaving (once cooled to ~55°C) and plates stored at 25°C for 1 d.

An initial screening experiment was conducted to identify presumptive ALAB that produce extracellular enzymes using A-retrograded mMRSA+1%SS. Previously selected LAB were resuscitated in Lactobacilli MRS broth (MRSB; BD Difco, United States). Those previously recovered at 15 or 45°C were cultured at 30 and 37°C, respectively, under anaerobic conditions for 24 h. Short streaks on mMRSA+1%SS were prepared and plates incubated under the same conditions but for 5 d. To visualize starch degradation, plates were placed over a petri dish bottom plate containing iodine crystals (IA005-100G; ChemSupply, Australia) for 1 min; the iodine vapors interacted with residual starch in the media resulting in a brown-purple color, depending on starch source and state. Isolates showing any degree of clearing in the colored iodine-starch complex surrounding growth (i.e., hydrolysis) were identified as presumptive ALAB and were selected for further assessment. Inability to degrade starch was concluded if the starch-iodine complex color was visible up to the edge of growth.

To develop a profile of starch degradative capacity for presumptive ALAB, extracellular amylolytic activity was assessed with respect to each starch source (PS, SS, WS) and physical state (A- and B-Retrograded and Raw). As a positive RSD control, the Type strain of *L. amylovorus*, NRRL B4540 (NRRL, Agriculture Research Service culture collection, United States), was utilized. Test strains and *L. amylovorus* NRRL B4540 were resuscitated anaerobically in MRSB at 37°C and cells (4,000 × *g*) were resuspended in phosphate buffered saline (~10^8^ cfu/mL). A 5 μL aliquot of each was spotted onto the surface of each mMRSA+1%S media in duplicate. After drying, plates were incubated as previously described. Starch degradation was visualized with iodine vapor (1–5 min) and zones of hydrolysis were graded according to the degree of clearing (complete or partial), measuring the radial distance of the zone from the edge of growth. Hydrolysis observed for the positive control was used as a benchmark for grading hydrolysis by test isolates.

### 2.3 Identification of ALAB isolates

The identity of (presumptive) ALAB isolates was determined by Sanger sequencing of the 16S rRNA gene. Isolates were resuscitated anaerobically on MRSA at 37°C for 72 h. Pure colonies were suspended in 1 mL sterile deionized water and the cells collected by centrifugation (13,000 × *g*/1 min). Cells were resuspended in 200 μL 5% Chelex-100 resin (Bio-Rad, United States) and heated at 100°C for 10 min. After centrifugation (12,000 rpm/2 min), the supernatants were collected, and the DNA quantified using a Qubit fluorometer (Invitrogen, United States). 16S rRNA genes were amplified by PCR in a reaction mix comprising template DNA (5 ng), 12.5 μL MyTaq 2X Mix (Bioline Reagents, United Kingdom), 1 μL 27F-4 forward (5′-AGA GTT TGA TCM TGG CTC AG-3′) and 1492R-1 reverse (5′-CGG TTA CCT TGT TAC GAC TT-3′) primers (25 mM), and molecular grade water up to a final reaction mix volume of 25 μL. Initial denaturation occurred at 94°C for 5 min, followed by 35 cycles of 94°C/30 s (denaturation), 50°C/45 s (annealing) and 73°C/1.5 min (extension). Final annealing occurred at 72°C for 12 min. PCR products were confirmed by gel electrophoresis. Amplicons (20 μL) were cleaned using 20 μL MagBio HighPrep PCR magnetic beads (MagBio Genomics, United States) according to the manufacturer's instructions for use in tubes, except DNA was eluted in 10 μL 10 mM Tris-HCl pH 8.0 with 50 mM NaCl (50°C). Cleaned DNA was quantified and a 50–100 ng DNA suspension in 11 μL molecular grade water was combined with 1 μL 27F-4 forward primer (10 μM) prior to sequencing (Australian Genome Research Facility, Australia). Returned DNA sequences were used as the query in a standard NCBI (National Center for Biotechnology Information) blastn search to identify the closest species match (>99%).

### 2.4 Identification of GH13 enzyme-encoding genes associated with starch degradation in *Lactobacillaceae*

#### 2.4.1 α-amylase-encoding *amyA*

The literature was reviewed to identify candidate GH13 enzyme-encoding genes confirmed to be specifically associated with extracellular RSD by *Lactobacillaceae*. A specific α-amylase-encoding gene, *amyA*, was selected given its observation in more than one species and genus, and homologous genes from additional *Lactobacillaceae* strains were sought using a standard NCBI blastn search. For matches identified from WGS, the associated NCBI RefSeq assembly was viewed and *amyA* was located using the search term “starch binding,” as annotated by the NCBI Prokaryotic Genome Annotation Pipeline. For matches that corresponded to a stand-alone gene, these were viewed on NCBI GenBank. The *amyA* DNA sequence was obtained for each strain. Information regarding identified *amyA* genes and their encoded proteins was collated from the CAZy database, NCBI GenPept, and the Kyoto Encyclopedia of Genes and Genomes (KEGG) (Kanehisa et al., 2023). Specifically, enzyme classification and the presence of associated CBMs were noted from CAZy, while GenPept was used to identify conserved domains annotated by the integrated conserved domain database (CDD; concise results) (Lu et al., 2020). No information related to identified strains was found via KEGG. Evidence of the RSD ability of the additionally identified strains was also sought via a literature search.

#### 2.4.2 Genes associated with starch degradation in *L. amylovorus* and *L. amylolyticus*

GH13-enzyme encoding genes associated with starch degradation in *L. amylovorus* and *L. amylolyticus* were also sought given these species were most prevalent in the sampled starch processing line and were demonstrated to be “moderately” amylolytic without *amyA*. *Lactobacillus amylovorus* (*n* = 12) and *L. amylolyticus* (*n* = 3) strains with a complete or chromosome-level RefSeq genome assembly (as at the 25th of February 2024) were selected for investigation. Annotated genes (NCBI) potentially involved in starch degradation were sought using relevant search terms; “starch,” “pullulan,” “amylase,” and “family 13.” Identified genes and key features were cataloged and the DNA and protein sequences were obtained. Where available, additional information regarding identified genes and their encoded protein was sought via CAZy and NCBI GenPept, as previously described. For strains cataloged in KEGG, matching genes were identified and gene orthology (i.e., KEGG ortholog; KO) and the protein domain motifs predicted by the Protein families database (Pfam) were recorded. Evidence of the RSD ability of all identified strains was sought via a literature search. Using *L. amylovorus* DSM 20531 (*aka* NRRL B4540) as a reference (RefSeq accession GCA_002706375.1), the DNA and protein sequences of the GH13 enzyme-encoding genes identified were queried in standard NCBI blastn and blastp searches (for up to 1,000 matches) to identify homologous genes and proteins from *Lactobacillaceae* other than from *L. amylovorus* and *L. amylolyticus*. Matches were considered homologous based on query cover (QC) and percent identity (PI) being ≥90%.

### 2.5 Primer development and validation

Primers for *amyA* and the three identified GH13 enzyme-encoding genes (*pul, gly1, gly2*) have not been previously published. A multi sequence alignment (MSA) of reference sequences was performed using MUSCLE and the default settings in UGENE (Unipro UGENE v50.0) (Okonechnikov et al., 2012). The MSAs were manually interrogated to identify regions suitable for primers (i.e., avoiding di-nucleotide repeats and homopolymeric regions) and that ideally spanned the entire gene. Primer quality (https://www.idtdna.com/calc/analyzer), specificity (NCBI Primer-BLAST), and *in-silico* amplification (NCBI Primer-BLAST) were assessed.

For primer validation, *L. amylovorus* NRRL B4540 was used as a positive control; it was resuscitated anaerobically in MRSB at 37°C for 40 h and DNA was extracted using the Qiagen DNeasy PowerFood Microbial Kit (Qiagen, Germany) and according to its Quick-Start Protocol, except for the bead beating step which was implemented using a Mini-Beadbeater-24 (Biospec, United States) for 1 min. DNA was eluted in 50 μL Solution EB prior to quantification as previously described. PCR reaction mixes comprised template DNA (5 ng), 12.5 μL 2X LongAmp HotStart Taq (New England Biolabs, United States), 1 μL of forward and reverse primers (10 mM), and nuclease-free water up to a final volume of 25 μL. For *amyA* amplification, initial denaturation occurred at 94°C for 60 s, followed by 30 cycles of 94°C/20 s (denaturation), 58°C/30 s (annealing), and 65°C/160 s (extension). Final annealing occurred at 65°C for 10 min. PCR products were confirmed by gel electrophoresis. The same approach was applied for the other genes, albeit using tailored PCR conditions; for *pul, gly1*, and *gly2*, annealing temperatures and extension times of 57, 55, and 55°C, and 155, 90 and 70 s, were used, respectively.

### 2.6 Screening of ALAB isolates for starch degrading genes

Phenotypically identified ALAB (Table 1) were selected for screening to determine if they had the starch degrading genes, *amyA, pul, gly1*, and *gly2*. Isolates were resuscitated anaerobically in MRSB at 30 (*L. rhamnosus* only) or 37°C for 40 h and DNA was subsequently extracted using the Qiagen DNeasy PowerFood Microbial Kit (Qiagen, Germany) as previously described. DNA previously extracted from *L. amylovorus* NRRL B4540 was used as a positive control in all assessments. Gene amplification was attempted using the validated PCR protocols and PCR products were confirmed by gel electrophoresis. Amplicons were stored at −20°C.

**Table 1:** Identity and starch degradation profile for LAB isolates from a starch processing line, compared to RSD *L. amylovorus* NRRL B4540.

**Species**	**ID**	**Amylolytic activity rating**	**Radial starch hydrolysis zone (mm) according to grade (complete-partial-partial/purple hydrolysis) for LAB isolates grown on different starch sources and states**								
			**A-Retrograded** ^a^			**B-Retrograded** ^b^			**Raw** ^c^		
			**SS**	**PS**	**WS**	**SS**	**PS**	**WS**	**SS**	**PS**	**WS**
*L. amylovorus*	1	Moderate	4-0-0	0-5-4	0-3-3	5-0-0	0-5-3	0-2-2	0-7-0	0-0-0	0-0-0
*L. amylovorus*	2	Moderate	3-0-0	0-3-3	0-2-3	5-0-0	0-3-2	0-2-2	0-7-0	0-0-0	0-0-0
*L. amylovorus*	3	Moderate	4-0-0	0-4-3	0-3-2	5-0-0	0-5-3	0-2-1	0-6-0	0-0-0	0-0-0
*L. amylolyticus*	1	Moderate	4-0-0	0-5-3	0-3-2	5-0-0	0-4-3	0-3-3	0-6-0	0-0-0	0-0-0
*L. amylolyticus*	2	Weak	1-0-0	0-0-2	0-0-2	1-0-0	0-0-2	0-0-0	0-3-0	0-0-0	0-0-0
*Limosilactobacillus reuteri*	1	Moderate	4-0-0	0-4-2	0-3-2	4-0-0	0-4-3	0-3-2	0-6-0	0-0-0	0-0-0
*L. reuteri*	2	Weak	1-0-0	0-1-3	0-1-2	2-0-0	0-1-3	0-1-2	0-3-0	0-0-0	0-0-0
*Lacticaseibacillus rhamnosus*	1	Negligible	0-0-0	0-0-1	0-0-2	0-0-0	0-0-2	0-0-1	0-0-0	0-0-0	0-0-0
*L. amylovorus*	NRRL B4540	Strong	3-12-0	4-12-2	4-11-2	3-11-0	4-12-3	4-11-2	2-16-0	0-1-0	5-0-0

^a^Starch added to media prior to autoclaving (gelatinization); starch retrograded at 25°C for 1 d prior to inoculation.

^b^Starch added to media prior to autoclaving (gelatinization); starch retrograded at 4°C for 3 d prior to inoculation.

^c^Starch added to media after autoclaving and cooling to 55°C; plates stored at 25°C for 1 d prior to inoculation.

### 2.7 Multi-amplicon sequencing of starch degradation-associated genes

Starch degrading gene amplicons (*pul, gly1*, and *gly2* only) detected in ALAB test isolates and the positive control were cleaned up using MagBio HighPrep PCR magnetic beads (0.8X) as previously described. Per test isolate/positive control (*n* = 6), multiple amplicons (2 or 3 genes per isolate/strain) were combined in equimolar (fmol) proportions to achieve a final DNA concentration of 200 fmol in 11.5 μL nuclease-free water. Using the ONT Native Barcoding Kit 24 V14 (SQK-NBD114.24) and recommended third-party reagents, the manufacturer's instructions for the Ligation sequencing amplicon—Native Barcoding Kit 24 V14 (https://community.nanoporetech.com/docs/prepare/library_prep_protocols/ligation-sequencing-amplicons-native-barcoding-v14-sqk-nbd114-24; accessed May 13, 2024), were followed to undertake the DNA end-prep, native barcode ligation and adapter ligation steps to prepare the DNA library for sequencing. A deviation from the protocol occurred during bead clean-up in the adapter ligation step where AMPure XP beads were added to the pooled DNA at 0.8X. The barcoded and pooled DNA library (~40 fmol in 12 μL elution buffer) was sequenced on an ONT flowcell (FLO-MIN114, R10.4.1), fitted to the MinION™ Mk1C, as per the manufacturer's instructions. MinKNOW software (version 24.02.16) was used for raw data collection. Sequencing ran until each barcoded sample reached at least ~13,000 reads (mean reads 15,989 ± 2,262 standard deviation; SD).

### 2.8 Data analysis workflow

The POD5 files generated during sequencing were basecalled, demultiplexed, trimmed (barcodes and adapters) and filtered to a minimum quality score of 10 using MinKNOW (version 23.11.7) with Dorado (version 7.2.13) in super accuracy mode, retaining reads with barcodes on one end. To sort the mixed amplicon reads into their respective *pul, gly1* (*L. amylovorus* only), and *gly2* gene groups, the ONT EPI2ME (desktop application version 5.1.10) wf-amplicon workflow was implemented in variant calling mode. The DNA sequences of *pul, gly1*, and *gly2* derived from *L. amylovorus* DSM 20531 (RefSeq accession GCA_002706375.1) and *L. amylolyticus* L5 (RefSeq accession GCF_003999355.1; *pul* and *gly2* only) were trimmed at the start (forward) and end (reverse) of primer binding sites, and were used as input reference sequences for alignment (Minimap2; version 2.26-r1175), variant calling (Medaka; version 1.11.1), and to generate a consensus sequence for *L. amylovorus and L. amylolyticus* test isolates, respectively. Reads between 1,300 and 3,500 bp and a quality score of ≥20 were selected, with the –drop_frac_longest_reads –take_longest_remaining_reads options disabled to instead perform random downsampling of 3,000 reads. All other parameters were maintained as per the default settings, including for variant calling where the model used was auto selected based on the basecaller configuration (dna_r10.4.1_e8.2_400bps_sup@v4.2.0). Output workflow reports and VCF files were used to collate information related to alignment sequence coverage and accuracy, as well as variants identified. Consensus sequence outputs were used for downstream analysis. Analysis of sequenced genes from the positive control with respect to reference gene sequences from *L. amylovorus* DSM 20531 served as an internal validation of sequencing accuracy and workflow performance.

### 2.9 Sequence similarity of starch degradation-associated genes and their deduced proteins

Consensus gene sequences were collated with trimmed (based on primer amplifiable region) gene sequences obtained for *L. amylovorus* and *L. amylolyticus* strains with published WGS (as per Supplementary Tables 2–4). A MSA was performed as previously described and the similarity (%) of each sequence with respect to each other was calculated in UGENE using the statistics function. Published strains with partial gene sequences were omitted from the analysis. Consensus sequences for test isolate genes were translated to deduced amino acid sequences with the Expasy translate tool (https://web.expasy.org/translate/) (Duvaud et al., 2021) using the standard genetic code. Given the entire gene sequence was not captured with the primers used and could therefore not resolve the entire open reading frame (ORF), the most likely deduced amino acid sequence (i.e., reading frame; RF) was selected based on similarity to the reference strains, as well as with consideration of premature stop codons and how these affected the number and length of coding regions. A MSA and similarity assessment was performed as previously described, and a manual assessment of frameshift mutations arising because of gene sequence variants was made.

### 2.10 Protein sequence homologs and domain annotation

Deduced amino acid sequences were queried by blastp to identify homologous proteins (QC and PI ≥ 90%) and to annotate the conserved domains (CDD concise results and specific hits) for comparison to published strains. Additional information regarding CAZy sub-family/CBM classification, EC number (top match) and enzyme substrate was sought via the dbCAN3 web server (https://bcb.unl.edu/dbCAN2/) (Zheng et al., 2023) using gene sequences as the input and HMMER:dbCAN-sub mode, whilst amino acid sequences were queried via the SignalP-−6.0 server (https://services.healthtech.dtu.dk/services/SignalP-6.0/) (Teufel et al., 2022) to predict (in slow mode) the presence of signal peptides and their cleavage site location. In each assessment, the whole gene and protein sequence of species-specific reference strains were used as the query to obtain a complete annotation; this would identify whether any domains or features were potentially missed for test isolates given the amplified region.

## 3 Results

### 3.1 Starch degrading capacity of *Lactobacillaceae* isolates

Of the 61 LAB isolates recovered from starch processing line samples, eight were selected as presumptive ALAB based on varying degrees of hydrolysis of A-retrograded SS in mMRSA (data not shown), which was also indicative of extracellular enzyme production. The identity of presumptive ALAB isolates was confirmed (Table 1), the majority of which are known to be innately amylolytic; *L. amylovorus* (*n* = 3) and *L. amylolyticus* (*n* = 2). Other identified isolates were *Limosilactobacillus reuteri* (*n* = 2) and *Lacticaseibacillus rhamnosus* (*n* = 1).

**Figure 1:**
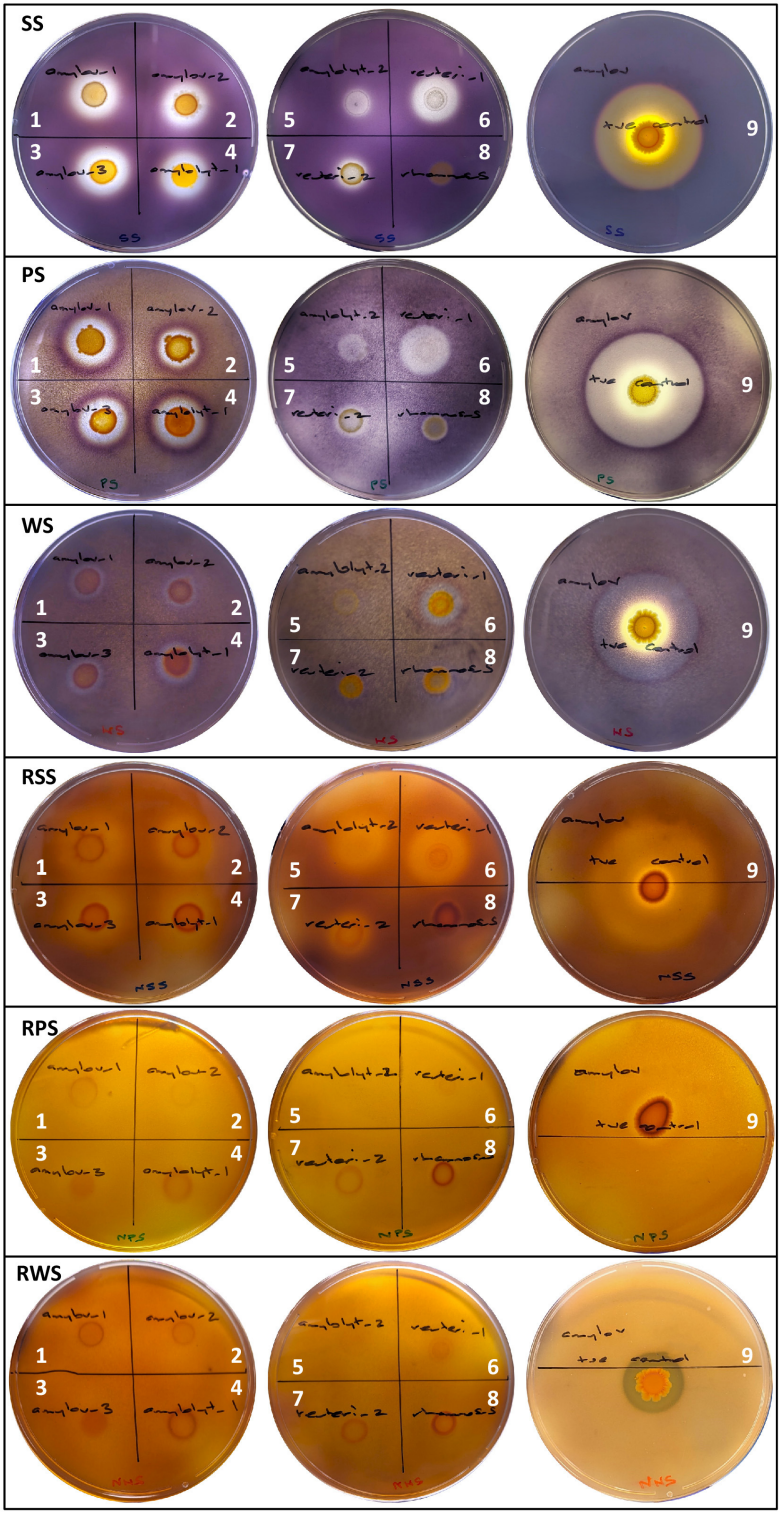
Starch hydrolysis by LAB isolates and *L. amylovorus* NRRL B4540 on mMRSA+1%S with retrograded and Raw, Soluble Starch, Potato Starch and Wheat Starch. Isolates are designated (1) *L. amylovorus* 1, (2) *L. amylovorus* 2, (3) *L. amylovorus* 3, (4) *L. amylolyticus* 1, (5) *L. amylolyticus* 2, (6) *L. reuteri* 1, (7) *L. reuteri* 2, (8) *L. rhamnosus* 1, and (9) positive control strain, *L. amylovorus* NRRL B4540.

A subsequent assessment was undertaken using three starch sources (PS, SS, WS) prepared in mMRSA to yield three physical starch states (A- and B-Retrograded and Raw) to examine hydrolysis. Using the amylolytic response of known RSD strain, *L. amylovorus* NRRL B4540, as a benchmark for assessing the response of test isolates (Table 1 and Figure 1), three grades of starch hydrolysis by extracellular enzymes were established: (1) complete starch hydrolysis with total clearing in starch granules; (2) partial hydrolysis with partially stained, diffuse starch granules still visible; and (3) partial-purple hydrolysis with darker, partially purple-stained, diffuse starch granules still visible (Figure 1). Confirmed ALAB isolates produced extracellular enzymes that hydrolyzed retrograded starches and raw SS to varying degrees, but the amylolytic activity of *L. rhamnosus* was rated negligible (1–2 mm partial-purple zones of hydrolysis observed only on retrograded PS and WS). All *L. amylovorus* isolates and *L. amylolyticus* 1 and *L. reuteri* 1 were similarly moderate in their hydrolytic activity, but weaker compared to the strong activity of *L. amylovorus* NRRL B4540, while overall amylolytic activity was rated weak for *L. amylolyticus* 1 and *L. reuteri* 2. Differences in response between the same species were observed, where *L. amylolyticus* 1 and *L. reuteri* 1 (moderate) were notably more amylolytic than *L. amylolyticus* 2 *and L. reuteri* 2 (weak). The response of the three *L. amylovorus* isolates was similar.

No test isolate was observed to produce an extracellular enzyme that could hydrolyze raw PS or WS, though partial hydrolysis of raw SS was observed (3- and 6–7-mm zones for weak and moderate ALAB isolates, respectively) (Table 1). This was in contrast to *L. amylovorus* NRRL B4540 which caused complete (2 mm) and partial (16 mm) hydrolysis of raw SS, complete (5 mm) hydrolysis of raw WS, and minimal, partial hydrolysis (1 mm) of raw PS. Hydrolytic activity on A- and B-Retrograded starches was largely indistinguishable for all isolates and will be considered collectively hereafter (including in Figure 1). The complete hydrolysis of retrograded starch was only seen for SS (1–2- and 3–5-mm zones for weak and moderate ALAB isolates, respectively), whilst partial and/or partial-purple zones of hydrolysis were otherwise observed for retrograded PS and WS, with partial zones ranging from 0–1 to 2–5 mm, and partial-purple zones ranging from 1–3 to 1–4 mm, for weak and moderate ALAB isolates, respectively (Table 1).

Based on the amylolytic response of test isolates and *L. amylovorus* NRRL B4540 (Table 1 and Figure 1), raw starches were measurably more resistant to enzymatic hydrolysis than retrograded starches. With regards to starch source, SS was most sensitive to hydrolysis, being the only starch source that was completely hydrolyzed by test isolates in its retrograded state, and the only raw starch source that was partially hydrolyzed. Based on test isolate activity, PS was marginally more sensitive to hydrolysis than WS, however raw WS was completely hydrolyzed by *L. amylovorus* NRRL B4540, whereas raw PS was only partially hydrolyzed.

### 3.2 *amyA* in *Lactobacillaceae*

A review of amylolytic LAB (Petrova et al., 2013) described strains of three species, *Lacticaseibacillus manihotivorans* (GenBank accession AF126051.1), *Lactiplantibacillus plantarum* A6 (GenBank accession U62095.1), and *L. amylovorus* CIP 102989 (GenBank accession U620926.1; = DSM 20531/NRRL B4540), that share a largely homologous (~98%) α-amylase-encoding *amyA* gene, the characterized protein sequences of which include an α-amylase catalytic domain as well as a unique SBD comprised of tandem repeats (× 4–5) of CBM26. In separate studies, RSD was confirmed for each strain (Imam et al., 1991; Giraud et al., 1994; Talamond et al., 2002), with the SBD confirmed as required to facilitate this (Rodriguez Sanoja et al., 2000). A blastn search identified four homologous regions/genes in other *Lactobacillaceae* (>95% QC and PI): *L. amylovorus* strains L4 (partial gene; RefSeq accession GCF_022642685.1) and 1394N20 (RefSeq accession GCF_021398395.1), and *L. plantarum* S21 (GenBank accession KJ440080.1). Although *amyA* has been independently characterized and sequenced from *L. amylovorus* strain CIP 102989, a homologous region from the whole genome of the same strain, albeit deposited as DSM 20531, was also identified by blastn (RefSeq accession GCF_002706375.1). Of the additional *L. amylovorus* strains identified, there was no evidence in the literature of their RSD ability, although their annotated protein sequences indicate the presence of the RSD-associated SBD with CBM26 tandem repeat units. Whilst the *amyA* gene of *L. plantarum* S21 shares 97% homology with that of *L. plantarum* A6, and also holds a SBD, it is unable to degrade raw starch (Kanpiengjai et al., 2015). However, this gene and its sequence was nevertheless included in subsequent primer development and sequence analyses for added diversity. A summary of the key features of *amyA* and its encoded protein as identified in published *Lactobacillaceae* species is presented in Table 2. Complete details for all strains are presented in Supplementary Table 1.

**Table 2:** Summary of attributes of starch degrading genes and their encoded proteins identified in select *Lactobacillaceae*.

**Gene/enzyme**	**Identified in (species)**	**# strains^a^**	**DNA/protein sequence length (bp/aa)**	**CAZy CBM family**	**GenPept protein designation**	**Protein domains CDD^b^**	**KO^c^**	**Protein domains Pfam^d^**
*amyA* α-amylase	*L. manihotivorans L. plantarum L. amylovorus*	1 2 4	2,733–3,392 901–971	26 (× 4–5)	Alpha-amylase or starch-binding protein	AmyAc_bac1_Amy, Aamy_C, CBM26	No strains described	N/A
*pul* Pullulanase	*L. amylolyticus L. amylovorus*	3 of 3 12 of 12	3,573–3,591 1,190–1,197	41, 48	Thermostable or Type I pullulanase or pullulanase	pulA_type1 super family, CBM41_ pullulanase, SlpA (× 2)	1,200	PUD (× 2), CBM_48, Alpha-amylase (× 2), Pullul_strch_ C, DUF3459, SlpA (× 2)
*gly1* Glycoside hydrolase 1	*L. amylovorus*	12 of 12	1,794 597	None	Amylopullulanase or GH13 family or α-amylase	AmyAc_CMD	1,208	Alpha-amylase, hDGE_amylase
*gly2* Glycoside hydrolase 2	*L. amylolyticus L. amylovorus*	3 of 3 12 of 12	1,722–1,725 573–574	34	Neopullulanase or GH13 family or α-glycosidase	AmyAC_CMD, Alpha-amylase_N	1,208	Alpha-amylase_N, Alpha-amylase, hDGE_amylase, GHL6, GHL10 (× 2) MJ1316 (*L. amylolyticus* only)

^a^For amyA: the number of strains according to each species that were identified to have a homologous amyA gene amongst all Lactobacillaceae in the NCBI database; Other genes: the proportion of strains with a complete or chromosome level WGS RefSeq assembly that were identified to have the gene.

^b^Protein domains annotated by the conserved domain database via NCBI. **AmyAC_bac1_AmyA** (Alpha amylase catalytic domain found in bacterial Alpha-amylases); **Aamy_C** (Alpha amylase C-terminal domain); **AmyAc_CMD** (Alpha amylase catalytic domain found in cyclomaltodextrinases and related proteins); **Alpha-amylase_N** (Alpha amylase, N-terminal ig-like domain); **CBM26** (Starch-binding module 26); **CBM41_pullulanase** (Family 41 carbohydrate-binding module from pullulanase-like enzymes); **pulA_type I super family** (Pullulanase, type I); **SlpA** (Surface layer protein A domain).

^c^KEGG orthology number: **KO1200** represents pullulanase (EC: 3.2.1.41); **KO1208** represents Cyclomaltodextrinase/maltogenic alpha-amylase/neopullulanase (EC:3.2.1.54, 3.2.1.133, 3.2.1.135).

^d^Protein domains annotated by the protein families database via KEGG. **Alpha-amylase** (Alpha amylase catalytic domain); **Alpha-amylase_N** (Alpha amylase, N-terminal ig-like domain); **hDGE_amylase** (Glycogen debranching enzyme, glucanotransferase domain); **CBM_48** (Carbohydrate-binding module 48-Isoamylase N-terminal domain); **PUD** (Bacterial pullulanase-associated domain); **Pullul_strch_C** (Alpha-1,6-glucosidases, pullulanase-type, C-terminal domain); **SlpA** (Surface layer protein A domain); **GHL6** (Hypothetical glycosyl hydrolase 6); **GHL10** (Glycosyl hydrolase-like 10); **DUF3459** (Domain of unknown function); **MJ1316** (RNA cyclic group end recognition domain).

### 3.3 GH13 starch degradation-associated genes in *L. amylovorus* and *L. amylolyticus*

Three GH13 enzyme-encoding genes associated with starch degradation were variably found in *L. amylovorus* (*n* = 12) and *L. amylolyticus* (*n* = 2) strains with complete and chromosome-level assembled genomes in RefSeq (Table 2). Complete details are presented in Supplementary Tables 2–4.

A putative pullulanase gene (*pul*) was detected in all *L. amylovorus* and *L. amylolyticus* strains (Table 2 and Supplementary Table 2). The encoded protein sequences were mostly designated as a Type I pullulanase in GenPept, but some were designated thermostable pullulanase or just pullulanase. Despite this, CDD protein annotation described the same domains which included a catalytic domain and CBM41 associated with pullulanases, and two adjacent surface layer protein A (SlpA) domains. Of the three *L. amylovorus* strains characterized in KEGG, *pul* was assigned to KO1200, representing pullulanase (EC: 3.2.1.41), all sharing the same Pfam-annotated protein domain structure which similarly included a pullulanase associated catalytic domain and two adjacent SlpA domains, as well as a CBM41. Whilst *L. amylolyticus* L6 is listed in KEGG (as the sole representative strain), *pul* is described as a pseudogene and the encoded protein is therefore not characterized. CAZy identified both CBM41 and CBM48 in *pul*-encoded proteins for the described strains.

The two other GH13 enzyme-encoding genes and associated proteins could not be conclusively defined due to variability in the GenPept designation for each protein/strain. Therefore, tentative, generic names were assigned: glycoside hydrolase 1 (*gly1*) and 2 (*gly2*), both of which are identified as enzymes involved in starch degradation. The *gly1* gene was only detected in the genomes of *L. amylovorus* (Table 2 and Supplementary Table 3) and the encoded protein sequences were variably designated as amylopullulanase, α-amylase or GH13 family by GenPept, with an α-amylase catalytic domain typical of cyclomaltodextrinases identified via the CDD. In KEGG, *gly1* was described for three strains and was assigned to KO1208, representing cyclomaltodextrinases (EC: 3.2.1.54), maltogenic α-amylases (EC: 3.2.1.133) and neopullulanases (EC: 3.2.1.135); an α-amylase catalytic domain and glycogen debranching enzyme domain were identified by Pfam. The *gly2* gene was detected in *L. amylovorus* and *L. amylolyticus* strains (Table 2 and Supplementary Figure 4). The encoded protein sequences were variably designated as neopullulanase, α-glycosidase or GH13 family by GenPept, with α-amylase catalytic domains identified via the CDD. In KEGG, *gly2* was described in three *L. amylovorus* and one *L. amylolyticus* strain and was assigned to KO1208 as for *gly1*. Whilst a CBM was not identified in the protein encoded by *gly1* in CAZy, CBM34 was identified in the protein encoded by *gly2* for described strains. No reports of RSD by the *L. amylovorus* and *L. amylolyticus* strains identified were found.

Using the gene and encoded protein sequences for *pul, gly1*, and *gly2* from *L. amylovorus* DSM 20531, BLAST searches revealed few additional *Lactobacillaceae* other than *L. amylovorus* and *L. amylolyticus* with genes or proteins that were considered homologous (i.e., QC and PI ≥ 90%). Homologous *pul* genes were identified in four *Lactobacillus crispatus* strains (QC 100% and PI 98.1%) whilst “Type 1 pullulanase” protein (Pul) matches (QC 100% and PI ≥ 92%) were similarly identified from *L. crispatus* (*n* = 13) and unclassified *Lactobacillus* spp. (*n* = 3). Whilst no homologous gene sequences were identified for *gly1* or *gly2*, generic GH13 proteins (QC ≥ 94% and PI ≥ 90%) were identified from unclassified *Lactobacillus* spp. (4X Gly1 and 8X Gly2) and *Lactobacillus kitasatonis* (4X Gly2).

### 3.4 Primer validation

Primers (Table 3) and PCR protocols for amplifying *amyA, pul, gly1*, and *gly2* were developed and validated using *L. amylovorus* NRRL B4540 as a positive control. Primers were designed to avoid problematic regions of the gene and were predicted to result in the amplification of variously truncated genes compared to the complete reference genes (Table 2). Validation demonstrated that all primers produced the expected sized bands (data not shown). Primer-BLAST confirmed amplification of the species/strains on which the primers were developed, as well as several strains of additional *Lactobacillaceae* (Table 3) amplified by the *pul* and *gly2* primers. *In-silico* amplification of *pul* from *L. crispatus* was consistent with blastn similarly identifying *pul* homologs in *L. crispatus*. Whilst *L. acidophilus* was not previously identified when searching for *gly2* homologs, these results suggest that select *L. acidophilus* strains have a gene that is partially homologous, consistent with the truncated region amplified by the developed primers.

**Table 3:** Primers for GH13 enzyme-encoding genes and their properties.

**Target gene**	**Primer**	**Sequence 5^′^ → 3^′^**	**GC (%)**	***T*_m_ (°C)**	**Amplicon length (bp)**	***In-silico* amplified species (*n*)^a^**
*amyA*	F-*amyA*	CGACATCAACTGATSACTCAAGC	47.8	56.0	2,585–3,084	*L. manihotivorans* (1) *L. plantarum* (2) *L. amylovorus* (3)
	R-*amyA*	GCAGCACATCAAGCAVTTCG	53.3	56.6		
*pul*	F-*pul*	GTGCATCAGCCATTTTGAGC	50.0	63.6	3,064–3,094	*L. amylolyticus* (3) *L. amylovorus* (12) *L. crispatus* (3)
	R-*pul*	CCAGTATTAGTGATGGAGCCA	47.6	62.1		
*gly1*	F-*gly1*	GGAGCAATTCAGGCCAATAC	50.0	62	1,712	*L. amylovorus* (12)
	R-*gly1*	AGGATGCTATMTTCACCGGA	47.5	62.4		
*gly2*	F-*gly2*	GGCTTACGASAATCKGGATC	52.5	61.8	1,375	*L. amylolyticus* (3) *L. amylovorus* (12) *L. acidophilus* (23)
	R-*gly2*	GCGTTCGTTTTCAYASTGCA	47.5	63.6		

^a^Using NCBI Primer-BLAST.

### 3.5 Detection of starch degradation-associated genes in ALAB isolates

The *pul, gly1*, and *gly2* genes were detected in *L. amylovorus* and *L. amylolyticus* test isolates, but not from *L. reuteri* or *L. rhamnosus* strains. This is not unexpected given that all our previous *in silico* assessments (blastn and PRIMER-BLAST) failed to identify or amplify homologous genes from either of these species. The *amyA* gene was not detected in any test isolate, however, to ensure this outcome was not due to unforeseen sequence variation in the reverse primer-binding site of the SBD of *amyA*, PCR was repeated using an alternate reverse primer (R-Δ*amyA*: 5′-CAC GTC CTT GAA TTG TAC CG-3′) that would result in amplification of the more conserved catalytic domain (~1,400 bp) of *amyA*. Using an adjusted and validated PCR protocol (annealing temperature of 56°C and extension time of 65 s), the truncated *amyA* gene (i.e., Δ*amyA*) was not detected in any test isolate confirming these isolates do not have an *amyA* gene.

### 3.6 Sequencing accuracy and EPI2ME wf-amplicon performance

The EPI2ME wf-amplicon workflow randomly downsampled 3,000 multi-amplicon reads for test isolates and the positive control and mapped these to their species-corresponding reference *pul, gly1*, or *gly2* gene sequences based on similarity (Table 4). Due to the uneven distribution of multi-amplicon reads arising during sequencing, the number of randomly downsampled reads mapped to each gene varied across test isolates, with an average (± SD) sample size of 1,108 ± 334 reads. The average (±SD) basecall accuracy of downsampled reads was 99.55 ± 0.03%. The mean coverage (>97%) and accuracy (>96%) of read alignment to reference sequences was high with ≤ 4 unmapped reads per isolate. By default, mapped reads were further downsampled for variant calling, with a maximum depth of 300X on a 150X per-strand basis; only one of 485 variants identified across all isolates had a read depth < 300X (complete variant details are shown in Supplementary Table 5).

**Table 4:** Read alignment, variant and sequence similarity summary for starch-degrading genes from test isolates compared to species-corresponding reference sequences.

**Isolate**	**Mean basecall accuracy (%)^a^**	**Gene**	**Randomly sampled reads**	**Median read length (bp)**	**Mean coverage (%)**	**Mean accuracy (%)**	**Variant type and number identified** ^**b**^				**Consensus sequence length (bp)**	**Similarity to reference (%)**
							**SNP**	**MNP**	**INDEL**	**Other**		
*L. amylolyticus* 1	99.57	*pul*	1,200	3,064	98.2	96.6	62	6	2	1	3,070	97
		*gly2*	1,800	1,374	99.6	99.0	4	0	0	0	1,375	100
*L. amylolyticus* 2	99.58	*pul*	1,500	3,077	98.0	96.2	68	8	4	0	3,076	97
		*gly2*	1,500	1,374	99.6	98.7	8	0	0	0	1,375	99
*L. amylolyticus* L5	N/A	*pul*	*L. amylolyticus* reference sequence								3,076^c^	N/A
		*gly2*	*L. amylolyticus* reference sequence								1,375^c^	N/A
*L. amylovorus* 1	99.55	*pul*	480	3,038	97.8	96.5	42	1	5	1	3,041	97
		*gly1*	1,400	1,686	99.6	97.3	7	0	5	0	1,691	98
		*gly2*	847	1,374	99.6	99.1	3	0	0	0	1,375	100
*L. amylovorus* 2	99.56	*pul*	801	3,071	97.3	97.3	48	2	1	0	3,076	98
		*gly1*	1,000	1,686	99.7	96.1	26	1	2	0	1,685	97
		*gly2*	1,200	1,370	99.4	99.1	2	0	0	0	1,375	100
*L. amylovorus* 3	99.54	*pul*	773	3,071	97.9	97.3	49	1	3	0	3,076	98
		*gly1*	1,000	1,686	99.7	96.1	25	1	3	1	1,686	97
		*gly2*	1,200	1,375	99.7	92.2	81	9	0	0	1,375	93
*L. amylovorus* NRRL B4540^d^	99.50	*pul*	999	3,080	98.6	99.2	0	0	0	0	3,085	100
		*gly1*	1,300	1,711	99.8	99.1	0	0	0	0	1,712	100
		*gly2*	726	1,367	99.3	99.1	3	0	0	0	1,375	100
*L. amylovorus* DSM 20531	N/A	*pul*	*L. amylovorus* reference sequence								3,085^c^	N/A
		*gly1*	*L. amylovorus* reference sequence								1,712^c^	N/A
		*gly2*	*L. amylovorus* reference sequence								1,375^c^	N/A

^a^Calculated based on mean Phred score of reads used for alignment; not applicable for reference sequences.

^b^SNP, single nucleotide polymorphism; MNP, multiple nucleotide polymorphism; INDEL, insertion or deletion; Other, variant not otherwise categorized.

^c^Length of trimmed reference gene sequence (i.e., primer-amplified region).

^d^Positive control isolate; aka *L. amylovorus* DSM 20531.

Prior to undertaking downstream analysis of test isolates, gene sequences from the sequenced positive control, *L. amylovorus* NRRL B4540, were compared to reference gene sequences derived from the published WGS of the same strain. Except for three single nucleotide polymorphisms (SNP) detected at the position of degenerate bases within the primer binding regions of *gly2*, all amplified gene regions were homologous (100% similarity) without any legitimate variants detected compared to their respective reference sequences. This, combined with an overall basecall accuracy of ≥99.5%, provided confidence in the reads and EPI2ME workflow outcomes with respect to test isolates.

### 3.7 Gene variant and similarity analysis

The similarity of test isolate (consensus) gene sequences to their (trimmed) species-corresponding reference sequences and other published strains was assessed by considering sequence length and similarity and identified variants. A summary of variants is provided in Table 4, whilst a comprehensive description of all variants is provided in Supplementary Table 5 as adapted from the VCF output from EPI2ME. Complete gene sequence similarity analysis is provided in Supplementary Table 6, determined from the MSAs (Supplementary Figures 1–3 for *pul, gly1*, and *gly2*, respectively).

The *pul* gene was the least conserved across test isolates and published strains, with species-based similarity and sequence length ranging 93–100% and 3,041–3,085 bp, respectively, as a result of various polymorphisms and INDEL events. Sequences from *L. amylovorus* 2 and 3 shared 100% similarity, with an identical 9 bp deletion event (position 3,051) and ~45 shared polymorphisms when compared to the reference strain (DSM 20531), to which there was 98% similarity. *L. amylovorus* 1 also shared the 9 bp deletion at position 3,051, which was revealed to occur in *pul* for 7 out of 11 published *L. amylovorus* strains. Three additional deletion events (18, 8, and 9 bp at position 172, 198, and 229, respectively) were recorded in *L. amylovorus* 1, the second of which was similar to a 9 bp deletion observed in strain CICC6090. Together with the 43 polymorphisms identified in *L. amylovorus* 1, these variations resulted in 97% similarity to the reference strain (DSM 20531) and 98–99% similarity with respect to the other *L. amylovorus* test isolates. Regarding *L. amylolyticus*, test isolates were both 97% homologous to the reference strain (L5) due to various polymorphisms (~70) and INDEL events, but only shared 96% similarity with respect to each other given that these variants, and especially the INDELS, were isolate specific. Due to several conserved, species-specific variations, similarity between species ranged 93–95%.

The *gly1* gene, only present in *L. amylovorus*, was highly conserved (99–100% similarity and 1,712 bp) across published strains, with the MSA indicating no INDEL events. In contrast, whilst test isolates were largely homologous to published strains (97–98% similarity), frequent polymorphisms and INDEL events were detected resulting in shorter, 1,685–1,691 bp, consensus sequences. Test isolates 2 and 3 shared 100% similarity, with two, almost identical deletion events (15–16 and 11 bp) occurring at the same positions (122/123 and 934, respectively), and 26 shared polymorphisms compared to the reference strain (DSM 20531). Test isolate 1 was more homologous (98% similarity and 7 SNP) to published strains but had a similar 15 bp deletion event to isolates 2 and 3 (at position 124) and had four additional, unique INDEL events ranging from 1 to 6 bp.

Except for one isolate, *gly2* was the most conserved gene across test isolates and published strains, with few polymorphisms and no INDELS detected. This resulted in a consistent sequence length of 1,375 bp for the amplified region and high similarity within species groups (99–100%). Test isolate *L. amylovorus* 3 was the exception with a unique sequence containing 90 polymorphisms in comparison to the reference sequence (DSM 20531), resulting in 93% similarity to this and other *L. amylovorus* isolates/strains. Whilst *gly2* was conserved within species groups, inter-species similarity was low (73–74%); this made it difficult to identify a universal primer set that would target both species.

### 3.8 Deduced amino acid sequence analysis

Test isolate genes were translated to deduced amino acid sequences and aligned to the trimmed protein sequences obtained for the published strains (Supplementary Figures 4–6 for Pul, Gly1, and Gly2, respectively). Sequence length and similarity were assessed (Supplementary Table 6), and the selected RF for the deduced amino acid sequences were interrogated to identify mutations (missense, nonsense/stop codon, and frameshift) arising from gene sequence polymorphisms and INDELS (Supplementary Tables 7–9 for Pul, Gly1, and Gly2, respectively).

Excluding sequences with premature stop codons (*L. amylovorus* 1 and *L. amylolyticus* L6), Pul sequence similarity and length ranged 90–100% and 1,020–1,027 aa, respectively, with greater sequence conservation observed within *L. amylovorus* (96–100%) and *L. amylolyticus* (93–97%) species groups, rather than between (90–91%). Although polymorphisms and INDEL events were detected in *pul* from *L. amylolyticus* 1 and 2 and *L. amylovorus* 2 and 3 test isolates, these did not result in frameshift mutations or stop codons and were therefore not predicted to have an impact on translation. However, for *L. amylovorus* 1, a stop codon at position 51 was predicted to interrupt translation. Translation of Pul for published strain, *L. amylolyticus* L6, was also predicted to be interrupted because of a deletion-precipitated stop codon toward the end of the sequence (position 833).

For translated *gly1* genes, initial RF selection for downstream analysis was complicated due to frameshift and missense mutations resulting in two potential RF candidates (+1 and +2) for *L. amylovorus* 2 and 3. Translation was predicted to be unlikely using either RF due to multiple stop codons, so RF+1 was selected to at least maintain a homologous alignment to all other sequences with respect to the N-terminal. With consideration of the whole sequence, test isolate Gly1 sequences were poorly aligned to published strains and to each other, especially *L. amylovorus* 2 and 3 which shared 57–60% similarity to published strains and only 20% similarity with each other due to variants in *gly1*. Test isolate *L. amylovorus* 1 was 91–92% homologous to published strains, and less similar to the other test isolates (54–57%). Nonetheless, translation of Gly1 was considered unlikely in all *L. amylovorus* test isolates. In contrast, Gly1 was highly conserved across published strains (99–100% similarity and 570 aa), without mutations predicted to affect translation.

The conservation of *gly2* across test isolates and published strains was maintained in deduced amino acid sequences, with a consistent length of 457 bp for the amplified region and high similarity within species groups (98–100%). For test isolate *L. amylovorus* 3, whilst the 90 polymorphisms in *gly2* resulted in only 93% similarity to other *L. amylovorus* isolates/strains, translated, these were largely silent (98% similarity). The sequence differences observed in *gly2* between species groups also translated to low inter-species similarity (76–77%) for Gly2. Overall, all Gly2 sequences were intact and predicted to be translatable.

### 3.9 Deduced protein domain annotation and homologs

Uninterrupted Pul sequences from test isolates were homologous with proteins described as Type I pullulanase, with matches identified from some *L. crispatus* strains in addition to those from the same species (Supplementary Table 7). Protein annotation revealed a consistent functional domain structure comprising a signal peptide cleaved by Signal Peptidase I (Sec/SPI at position 19|20), CBMs (CBM41 and CBM48) and an α-amylase catalytic domain associated with pullulanases (AmyAc_pullulanase_LD-like). dbCAN3 classified Pul into GH13_14 and EC 3.2.1./3.2.1.41 sub-families (comprising α-amylase and pullulanase activities), predicting starch as a substrate. Signature residues associated with the active, catalytic and binding sites of these domains were also present (CDD annotation). This protein structure was consistent with the reference strains, however, the SlpA domains typical of Pul in these species was not captured due to amplification of truncated genes. As previously mentioned, the translation of Pul was predicted unlikely in *L. amylovorus* test isolate 1 due to a premature stop codon. Whilst a subsequent ORF (from position 286) preserved the catalytic domain (and associated site residues), it excluded the annotated signal peptide and CBM41 domain, with potential truncation of CBM48 (based on position annotated by CAZy).

Gly1 in published *L. amylovorus* strains was primarily described as a generic GH13 protein with homologs restricted to the same species and *Lactobacillus* spp. (Supplementary Table 8). The dbCAN3 annotation of a domain aligned with the GH13_39 sub-family (comprising α-amylases and pullulanases) overlapped with the CDD annotation of an α-amylase catalytic domain (AmyAC_CMD) typical of cyclomaltodextrinases. The reference strain was further annotated by dbCAN3 with an N-terminal CBM34 domain predicted to bind to starch, but this was not annotated in test isolate sequences, potentially due to truncated amplification omitting the first 16 residues and/or because of INDEL-encoded mutations. No signal peptide was predicted.

Homologs of Gly2 were described as generic GH13 proteins, and with consideration of the full-length protein (reference strains), were largely identified from the same species (Supplementary Table 9). Despite sequence dissimilarity, protein annotation was consistent across *L. amylolyticus* and *L. amylovorus* isolates/strains, classifying Gly2 from the GH13_20 (comprising α-amylase, maltogenic α-amylase, neopullulanase, pullulanase and cyclomaltodextrinase) and EC 3.2.1.54 (cyclomaltodextrinase) sub-families. CDD-annotation identified an N-terminal α-amylase-associated domain (Alpha-amylase_N) which is further associated with an uncharacterized superfamily domain (X25_BaPul) described as potentially representative of CBMs in pullulanases. dbCAN3 annotation identified CBM34 for the same region, predicting starch as the substrate. The same cyclomaltodextrinase-associated α-amylase catalytic domain found in Gly1 was also found in the C-terminal of Gly2. No signal peptide was predicted.

## 4 Discussion

Investigating the starch degrading potential of LAB has been an area of ongoing interest since the first species were described (Nakamura and Crowell, 1979; Nakamura, 1981), and the development of experimental approaches that enable efficient yet informative assessments of ALAB-associated traits remains important. With this in mind, we explored the utility of diversified starch agar plate assays to assess extracellular enzyme activity of LAB in response to raw and retrograded starches, alongside a targeted GH13-enzyme encoding gene assay that enabled the examination of near complete starch-degrading gene sequences to infer probable enzyme functionality.

Amylolytic LAB that produce extracellular enzymes capable of RSD below starch gelatinization temperatures are desired (Petrova and Petrov, 2012; Moradi et al., 2014; Sun et al., 2015; Božić et al., 2017; Roy et al., 2021). Whilst the inclusion of SS in starch agar plate assays is typical (Kim et al., 2008; Pascon et al., 2011; American Society for Microbiology, 2012; Fossi and Tavea, 2013), the hydrolysis of such gelatinized SS offers a limited view of amylolytic potential given its structure is more sensitive compared to that of raw starch (Giraud et al., 1994; Jane, 1995). Retrogradation occurs with the cooling of gelatinized starch, increasing resistance somewhat, but is less resistant compared to raw starch (Wang et al., 2015). The extent of retrogradation has been reported to increase with storage time and at cooler temperatures (Jane, 1995; Wang et al., 2015).

On this basis, we varied the physical state of starch in mMRSA+1%S and hypothesized that starch would become increasingly susceptible to hydrolysis moving from raw starch (not autoclaved) to B-Retrograded (autoclaved and stored at 4°C/3 d) and then to A-Retrograded (autoclaved and stored at 25°C/1 d) starch states. Raw starch was confirmed most resistant, with the *L. amylovorus* control (NRRL B4540) the only strain capable of complete RSD, and no discernable difference was noted in the hydrolysis of A- and B-Retrograded starches. The rapid differentiation of isolate amylolytic potential on the basis of starch state alone highlighted the utility of this diversified approach.

The inclusion of different starch sources provided additional utility, with variation in the size and appearance of zones of hydrolysis of PS, WS and SS offering clues regarding the versatility of the produced enzyme(s) for different substrates, and, potentially, their mode of action/identity. A systematic screening approach using starch agar plates with varying starch sources, components and substrates was previously implemented (Moradi et al., 2014) to identify high amylolytic enzyme-producing *Bacillus* species from industrial settings, which was verified to be as informative as quantitative analyses assessing total reducing sugars. In addition to attributing the size of zones of hydrolysis to enzyme productivity, the clarity and color of zones were used to hypothesize the enzyme(s) involved. The interpretation of zone appearance stems from the color differences of iodine-substrate complexes depending on the degree of polymerization of the substrate (Saibene et al., 2008; Pesek and Silaghi-Dumitrescu, 2024). Moradi et al. (2014) attributed red-colored zones on starch and amylopectin agar to α-amylase activity producing dextrin via the hydrolysis of α-1,4 glycosidic linkages only, and explain that complete hydrolysis (i.e., clear zones) requires an enzyme or a combination of enzymes that additionally target α-1,6 linkages. Starch comprises amylose and amylopectin polymers which form α-1,4-linked linear chains (amylose and amylopectin) and α-1,6-linked branches (amylopectin), so the complete hydrolysis or depolymerization of starch to its glucose sub-units requires a multi-target enzyme or a combination of complementary enzymes (Hii et al., 2012; Božić et al., 2017).

On retrograded mMRSA+1%S, we described variability in the clarity and color of zones of hydrolysis surrounding ALAB growth depending on starch source. Complete clarity was only observed with SS and therefore suggests enzymatic activity targeting both α-1,4 and 1,6 linkages. However, PS and WS sources were partially, and variably hydrolyzed, with zones presenting with degrees of starch granule diffusivity. The type of enzymatic activity ascribed to these partial zones of hydrolysis is inconclusive; that is, it is unclear if the varied diffusivity/color of residual starch granules is indicative of different starch hydrolysis products or simply indicative of a transitory state of hydrolysis where the same enzyme(s) that hydrolyzed SS are working similarly, but less efficiently, to hydrolyze the comparatively more resistant PS and WS. And with continued incubation, these partial zones could eventually become clear, a consideration for future assay development. The structure of starch varies considerably depending on botanical source (Hii et al., 2012; Božić et al., 2017), and whilst a consensus has not been reached, studies have reported that amylose is located within the center of starch granules (Božić et al., 2017). Since amylose tends to visibly complex with iodine more so than amylopectin, the darker purple rings we observed at the extremities of the hydrolytic zone on PS and WS could suggest the beginnings of enzymatic degradation, with a temporary increase in iodine complexing with more exposed amylose chains. This aligns with the idea of continued production of extracellular enzyme(s) that slowly diffuse through the agar, reaching further as a function of enzyme productivity. With respect to differences in amylolytic activity depending on retrograded WS or PS sources, whilst the pattern of the degradation zones was similar, activity was stronger targeting PS based on greater clarity and size of zones of hydrolysis. Given the B-pattern (double helices arranged in a monoclinic array), crystalline structure of tuber starches are described as “more open,” this could imply PS is more efficiently hydrolyzed, or more sensitive to degradation, than A-pattern (hexagonal array) starches like WS (Saibene et al., 2008). Conflicting observations were made on raw starch media where complete RSD by *L. amylovorus* DSM 20531 was only observed on mMRSA+1%WS; separate from the inherent resistance of different starch sources, this may be more explicitly indicative of enzyme-substrate specificity and/or the involvement of other enzymes.

The analysis of amino acid sequences, including those deduced from sequenced genes, has underpinned the characterization of starch-degrading enzymes produced by LAB (Petrova and Petrov, 2012; Petrova et al., 2013). Due to a lack of homology in gene sequences that encode amylolytic enzymes across *Lactobacillaceae*, GH13-enzyme-encoding genes conserved amongst *L. amylovorus* and *L. amylolyticus* (i.e., *gly1, gly2*, and *pul*) were selected to demonstrate our targeted multi-amplicon sequencing approach and subsequent analysis framework. Therefore, there was no expectation that these genes would be detected in our *L. reuteri* and *L. rhamnosus* test isolates, and this was confirmed. As such, further assessment with regard to these test isolates was not within the scope of this study. In addition to the conserved genes selected, extracellular, α-amylase+SBD-encoding *amyA* was also included given it is found more broadly, albeit rarely, across *Lactobacillaceae*, and because of the RSD phenotype that accompanies it. However, this gene was not detected in any of our test isolates, but was confirmed in the control, *L. amylovorus* NRRL B4540. *In silico, amyA* was only annotated in two of the 11 published *L. amylovorus* strains (L4 and 1394N20) examined here and was absent in *L. amylolyticus*, suggesting that *amyA* is not conserved across these species, further supporting its sporadic distribution in *Lactobacillaceae*, potentially reflective of evolutionary, environmental and/or genetic exchange drivers. Nevertheless, with near-complete DNA sequences for *gly1, gly2*, and *pul* detected from *L. amylovorus* and *L. amylolyticus* test isolates, we leveraged deduced amino acid sequences and predicted functional domains to develop hypotheses regarding the putative role of their encoded-enzymes in starch degradation and in the starch hydrolysis patterns observed in this study.

**Figure 2:**
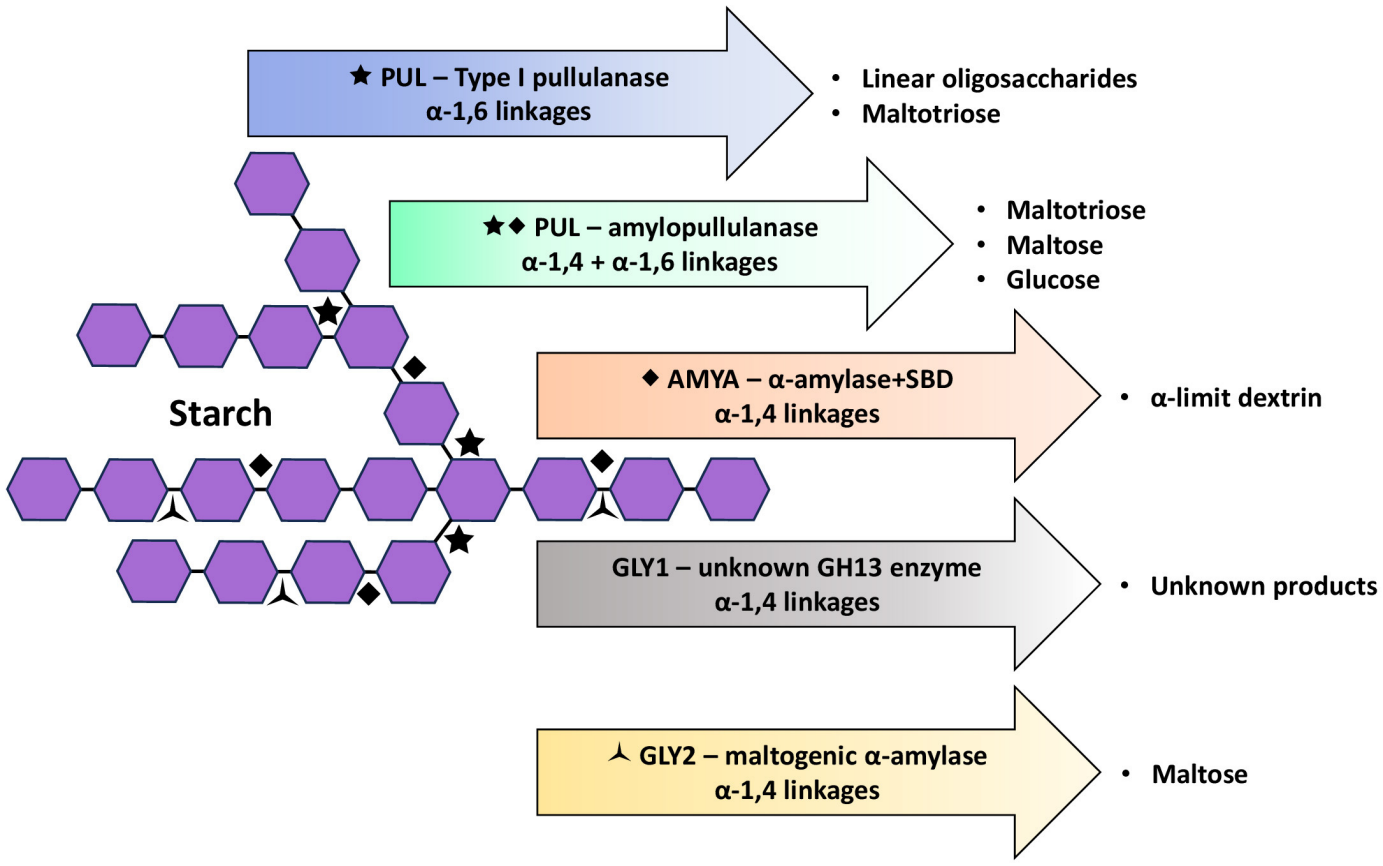
Glycosidic bond targets and primary products of starch hydrolysis by PUL (functioning as either a Type I pullulanase or an amylopullulanase), AMYA (an α-amylase with a starch-binding domain, SBD), GLY1 (unknown GH13 enzyme) and GLY2 (as a maltogenic α-amylase) (Hii et al., 2012; Drula et al., 2022; Zhang et al., 2022; Hutabarat and Stevensen, 2023; Naik et al., 2023).

The predicted functional domains deduced from *gly1* and *gly2* comprise an α-amylase catalytic domain that is typically identified in cyclomaltodextrinases (AmyAC_CMD), but which is also associated with neopullulanases and maltogenic amylases, each targeting α-1,4 linkages that variably produce maltose from cyclomaltodextrins and starch, or panose from pullulan (Lu et al., 2020). Additional dbCAN3 annotation suggests a CBM34 module in the N-terminal, which is typically associated with bacterial enzymes that target cyclodextrins (Drula et al., 2022). For Gly1, dbCAN3 assigned the catalytic domain to GH13_39; a sub-family associated with amylopullulanases described from *Alicyclobacillus, Bacillus* and *Thermoanaerobacterium* species in CAZy (Drula et al., 2022). Closer examination of amylopullulanase from one example (*Bacillus* XAL601) reveals a multi-functional-domain protein which shares AmyAc_CMD in common with Gly1 but is otherwise dissimilar. Without similar, previously characterized proteins to compare to Gly1, function beyond likely hydrolysis targets cannot be attributed within the scope of this study (Figure 2). Regardless, the contribution of Gly1 to starch degradation by test isolates here was ruled out given *gly1* is not found in *L. amylolyticus* species broadly, and because translation was likely impaired in our *L. amylovorus* test isolates. For Gly2, however, the assignment of the catalytic domain region to GH13_20 reveals a maltogenic α-amylase characterized in *L. amylolyticus* L6 (Zhang N. et al., 2023) that shares 99–100% similarity with our *L. amylolyticus* test isolate sequences. Therefore, Gly2 is putatively identified as a maltogenic α-amylase (EC 3.2.1.133) in our *L. amylolyticus* isolates; an intracellular enzyme that preferentially targets α-1,4 linkages in β-cyclodextrin, producing maltose, but which shows less hydrolytic activity for starch and negligible activity toward amylopectin, both suggesting this maltogenic α-amylase does not hydrolyze α-1,6 linkages (Zhang N. et al., 2023) (Figure 2). Because Gly2 sequence similarity between *L. amylolyticus* and *L. amylovorus* is 73–74%, we cannot be certain that Gly2 in *L. amylovorus* is also a maltogenic α-amylase. However, the residues identified as important in the conserved regions, substrate binding and transglycosylation sites of the maltogenic α-amylase produced by strain L6 (Zhang N. et al., 2023) are also present in Gly2 from *L. amylovorus* test isolates and published strains. Gly2 was predicted to function as an intracellular enzyme in published and test isolates on the basis that a signal peptide was not detected.

Type I pullulanases are debranching enzymes that hydrolyze the α-1,6 linkages in pullulan and branched polysaccharides (e.g., amylopectin in starch) to yield maltotriose and linear oligosaccharides, respectively (Hii et al., 2012; Naik et al., 2023) (Figure 2). Without specificity toward α-1,4 linkages, they can only partially hydrolyze starch. To our knowledge, the production of pullulanase by *L. amylovorus* or *L. amylolyticus* has not been previously addressed, however, genome annotation of several published strains here revealed a putative Type I pullulanase encoded by *pul*, which was similarly present in our test isolates, albeit with some sequence variation. Irrespective of the strain, Pul was annotated with a multi-domain structure predicted to target starch and which resembled those generally described for pullulanases (Naik et al., 2023); i.e., an N-terminal CBM41 followed by a catalytic domain in the C-terminal. In addition, Pul was annotated with a second CBM (family 48) located upstream of the catalytic domain, and an N-terminal Sec/SPI signal peptide predictive of extracellular secretion. Whilst not captured in the truncated sequences of test isolates, the tandem SlpA domains conserved across published strains (except the “pseudogene” in strain L6) represents an interesting feature that is thought to facilitate the attachment of secreted enzymes to the cell surface (Møller et al., 2017; van der Veer et al., 2019).

In two recent studies investigating α-glucan (Møller et al., 2017) and glycogen (Zhang et al., 2022) degradation by human gut and vaginal microbiota, putative, extracellular, pullulanase Type I enzymes produced by *L. acidophilus* NCFM (*La*Pul13_14; GenPept: APT18363) and *L. crispatus* L49 (GlgU; GenPept: RXF54749), respectively, were linked. These enzymes share the same multi-domain structure as has been annotated for Pul (data not shown), and further comparison reveals that *La*Pul13_14 (1,185 aa) and GlgU (1,251 aa) respectively share 65% and 52–53% similarity to Pul from our *L. amylovorus* DSM 20531 (1,197 aa) and *L. amylolyticus* L6 (1,193 aa) reference strains. Interestingly, *La*Pul13_14 and GlgU vary in their hydrolysis target, the former found to only target α-1,6 linkages in short-branched α-glucans (i.e., confirmed pullulanase Type I), whilst GlgU was capable of hydrolyzing α-1,4 and α-1,6 linkages in glycogen as well as soluble starch (Møller et al., 2017; Zhang et al., 2022). Both GlgU and *La*Pul13_14 have a Sec/SPI signal peptide conferring secretion, however, free enzyme in the supernatant was only detected for GlgU (Zhang et al., 2022). This could suggest that SlpA does not necessarily confer cell attachment of all the enzyme that is secreted, with some enzyme available in the extracellular environment. These defining differences may be associated with differing ecological pressures; as a gut microbe, *L. acidophilus* NCFM does not require *La*Pul13_14 to additionally target α-1,4 linkages given this can be fulfilled by human α-amylase during the digestion of starch-rich foods. However, in the absence of host-produced enzymes in the glycogen-rich vaginal environment, production of enzymes that can target both α-1,4 and α-1,6 linkages provides an ecological advantage, as is the case for *L. crispatus*. Pullulanases with dual targets are classed as Type II and are referred to as amylopullulanase or α-amylase-pullulanase, depending on the number of active sites (Nisha and Satyanarayana, 2013), and primarily yield maltotriose from starch (Hii et al., 2012) (Figure 2). Amylopullulanases have been additionally characterized from other LAB, from family GH13_14 in CAZy (Drula et al., 2022); vaginal strains *L. crispatus* MV-1A-US (GenPept: EEU28204) and *Lactobacillus iners* LEAF 3008A-a (GenPept: EFQ51965), and *L. plantarum* L137 (GenPept: BAF93906) isolated from a naturally fermented dish containing rice (Kim et al., 2008). A RSD α-amylase-pullulanase, putatively assigned to GH13_41, has also been described from starch industry waste isolate, *Amylolactobacillus amylophilus* GV6 (GenPept: APT18363) (Vishnu et al., 2006). Of these, amylopullulanase from *L. crispatus* MV-1A-US (1,251 aa) has the same domain structure as Pul, sharing 48–49% similarity, whilst the others are less similar (7–11%), all variably composed of additional CBMs and/or other functional domains (Zhang et al., 2022).

With consideration of the collective findings of the phenomic and genomic assessments of *L. amylovorus* and *L. amylolyticus* isolates/strains (Table 5), and within the scope of the starches and genes assessed here, we attempted to derive an association between the observed phenotype of isolates and these starch-degrading genes. Complete RSD was only observed for the control, *L. amylovorus* NRRL B4540, and was made in parallel with the detection of all genes assayed. If we assume Gly1 and Gly2 are intracellular only, and that complete starch degradation requires enzymes that target α-1,4 and α-1,6 linkages, the RSD phenotype observed for *L. amylovorus* NRRL B4540 would most likely be attributed to AmyA (α-1,4) and Pul (α-1,6). For *L. amylovorus* and *L. amylolyticus* test isolates, we attributed complete hydrolysis of retrograded SS (and possible, in-progress, complete hydrolysis of retrograded PS and WS) to enzymatic activity targeting both α-1,4 and α-1,6 linkages. In the absence of AmyA or Gly1, and with the continued assumption that Gly2 does not function extracellularly, Pul would remain the only enzyme attributable to the observed phenotype and might suggest the dual enzymatic activity of an amylopullulanase; an enzyme not previously described in these species. Indeed, dbCAN3 classification of Pul into EC sub-families associated with α-amylase (α-1,4) and pullulanase (α-1,6) targeting starch, supports this. Further, as previously discussed, there is precedent demonstrating that ecological pressures can drive the functional diversification of “pullulanases” to additionally target α-1,4 linkages (Zhang et al., 2022). Certainly, a starch processing line with a predominant LAB microbiome may select for ALAB with such dual activity. However, in addition to this hypothetical role of Pul, we must also consider an alternative scenario where enzymes predicted to function intracellularly, may be present extracellularly as a result of non-canonical secretory pathways (Liu and Bhunia, 2024) or cell lysis. In this scenario, if Gly2 was present extracellularly, at an appropriate concentration and with suitable diffusivity, it could function in tandem with Pul to break down α-1,4 and α-1,6 linkages, respectively. In this scenario, Pul functioning as an amylopullulanase would not necessarily be required.

**Table 5:** Starch-degrading phenotype and associated GH13 genes/predicted enzymes of *L. amylovorus* and *L. amylolyticus*.

**Isolate**	**Extracellular starch-degrading activity**		**Gene detection and predicted protein translatability** ^**a**^							
	**RSD**	**Rating**	***amyA***	**amyA**	***pul***	**Pul**	***gly1***	**Gly1**	***gly2***	**Gly2**
*L. amylolyticus* 1	No	Moderate	X	N/A	✓	✓	X	N/A	✓	✓
*L. amylolyticus* 2	No	Weak	X	N/A	✓	✓	X	N/A	✓	✓
*L. amylovorus* 1	No	Moderate	X	N/A	✓	X	✓	X	✓	✓
*L. amylovorus* 2	No	Moderate	X	N/A	✓	✓	✓	X	✓	✓
*L. amylovorus* 3	No	Moderate	X	N/A	✓	✓	✓	X	✓	✓
*L. amylovorus* NRRL B4540	Yes	Strong	✓	✓	✓	✓	✓	✓	✓	✓

^a^Green shading indicates where a gene was detected and the deduced protein was predicted to translate.

An association between phenotype and GH13 enzyme-encoding genes was also unclear when comparing outcomes for test isolates. Extracellular amylolytic activity was observed in *L. amylovorus* 1 despite INDEL induced changes predicting a truncated Pul protein without a signal peptide and N-terminal CBM41, and which suggested translation may not occur at all. In contrast, *pul* in all other isolates was predicted to encode both functional regions, yet most of these isolates were equally “moderate” in their extracellular starch-degrading activity. Whilst this cannot be explained within the scope of this study, the involvement of genes encoding alternate, extracellular starch-degrading enzymes cannot be ruled out for these previously uncharacterized wild strains, although a review of all CAZymes annotated in *L. amylovorus* DSM 20531 (Drula et al., 2022), for example, does not suggest any (data not shown). Alternatively, without isolating the precise function of each domain in Pul, including SlpA which was not captured by PCR and sequencing here, it is unclear if the truncated Pul in *L. amylovorus* 1 may still have residual function. A similar, unattributable difference in activity was observed between *L. amylolyticus* 1 and 2, despite Pul being intact in both. Others have shown that even a few residue substitutions can affect starch degrading activity by ALAB (Rodríguez-Sanoja et al., 2009; Petrova and Petrov, 2012), and larger deletion events in the signal peptide, for example, can diminish and even prevent substrate degradation (van der Veer et al., 2019). With regards to Pul in *L. amylolyticus* 2, there are numerous residue substitutions (>60) as well as a deletion (1 residue) and insertions (1 and 2 residues) compared to *L. amylolyticus* 1; one or more of these could be associated with the observed phenotypes depending on position with respect to the functional domains and associated binding, active and catalytic sites. Given the inherent limitations of the current screening tools to definitively characterize the independent and collaborative roles of gene-encoded enzymes in starch degradation, alternative approaches would be required to address these gaps, and to establish a genomic basis for starch-degrading phenotypes. To independently assess the starch-degrading activity of enzymes of interest (e.g., Pul more generally and Gly2 in *L. amylovorus*), substrate specificity studies using purified proteins, along with enzyme activity assays, would be essential. Additionally, to confirm gene expression, enzyme production, and starch hydrolysis products, transcriptomic, proteomic, and metabolomic analyses, respectively, would be necessary. Finally, to confirm the possible contribution of other amylolytic enzymes in test isolates, WGS would be required in the first instance to identify any novel genes.

Whilst confirming a genetic basis for starch-degrading phenotype is outside the scope and intention of our described screening tools, Nanopore-facilitated sequencing of near-complete amylolytic genes identified numerous gene and (deduced) protein sequence variants within the same species, suggesting our test isolates are unique and likely represent different strains. The greatest diversity in protein (and gene) sequences was observed in Pul from test isolates and published strains alike and could suggest, as we have hypothesized for our own isolates, niche adaptation. The majority of *L. amylovorus* published strains were isolated from animal feces and gastrointestinal tracts, whilst *L. amylolyticus* strains originated from fermented foods and beverages; it remains to be understood whether these Pul variants differ in their glycosidic linkage targets and/or starch degrading phenotype, and whether they are indeed Type I pullulanases or amylopullulanases. Certainly, others have shown that changes in the environment and/or microbiome can lead to sequence variation to suit (Møller et al., 2017; Stefanovic and McAuliffe, 2018; van der Veer et al., 2019; Hertzberger et al., 2022). In contrast, hypothetical, maltogenic α-amylase, or Gly2, was highly conserved within species groups and could suggest a more generic, or secondary role in starch degradation that is perhaps not subject to the same ecological pressures. Lastly, the detection of three defunct Gly1 variants in *L. amylovorus* test isolates was inconsistent with those from published strains in which Gly1 was highly conserved and intact. Similar to ecological pressures giving rise to evolved enzymes that will ensure survival, a lack of pressure to perform certain functions can render them obsolete and lead to gene decay or conversion to pseudogenes (Petrova et al., 2013; Velikova et al., 2016; Stefanovic and McAuliffe, 2018). This may explain the absence of this gene in *L. amylolyticus* more broadly.

This study describes a targeted and efficient methodological and analysis framework that harnesses complimentary phenomic and genomic assays informative of the starch degrading potential of *L. amylovorus* and *L. amylolyticus*, and specifically, RSD as a result of extracellular enzymatic activity. An adapted starch agar plate assay to include raw and diversified botanical starch sources facilitated differentiation of amylolytic capacity on the basis of extracellular enzyme productivity (size of clearing), efficiency (partial or complete clearing) and activity/specificity for different targets (i.e., starch state, source and glycosidic bond type). When combined with long-read, multi-amplicon sequencing-facilitated gene and deduced amino acid sequence analysis, extracellular RSD by a *L. amylovorus* control strain was hypothesized to be attributed to AmyA, a rare α-amylase with unique SBD, but which was predicted to also require the starch debranching activity of pullulanase (Pul) to completely degrade starch. This combination of enzymes is rare amongst *L. amylovorus*, and unapparent in *L. amylolyticus*, and was not detected in the wild strains examined here. In the absence of AmyA in our wild strains, Pul was assumed associated with complete hydrolysis of retrograded SS on plates; either as an amylopullulanase with dual glycoside hydrolase activity, or as a pullulanase working synergistically with Gly2, assuming Gly2 may be present extracellularly. Whilst further work would be necessary to characterize these enzymes, including those encoded by gene variants in our wild strains, these technologies, tools and bioinformatic analyses have provided the necessary evidence to warrant this. Further, this framework is likely adaptable for the direct analysis of LAB-rich microbiomes for amylolytic potential, and for the targeted screening of various other functions across different taxa.

## Data Availability

The data presented in this study are deposited in an online repository. The repository can be found at https://www.ncbi.nlm.nih.gov/bioproject/1237504, with BioProject accession number PRJNA1237504.
