# Supplementary material for: Long-read, multi-amplicon sequencing to explore genetic diversity associated with starch degrading phenotypes in amylolytic Lactobacillaceae
Source: Front Microbiol. 2025 Mar 26;16:1548052. doi: 10.3389/fmicb.2025.1548052 (PMC11980422; doi:10.3389/fmicb.2025.1548052)

**Supplementary Figure 1.** MSA of *pul* DNA sequences from test isolates and reference strains (trimmed).


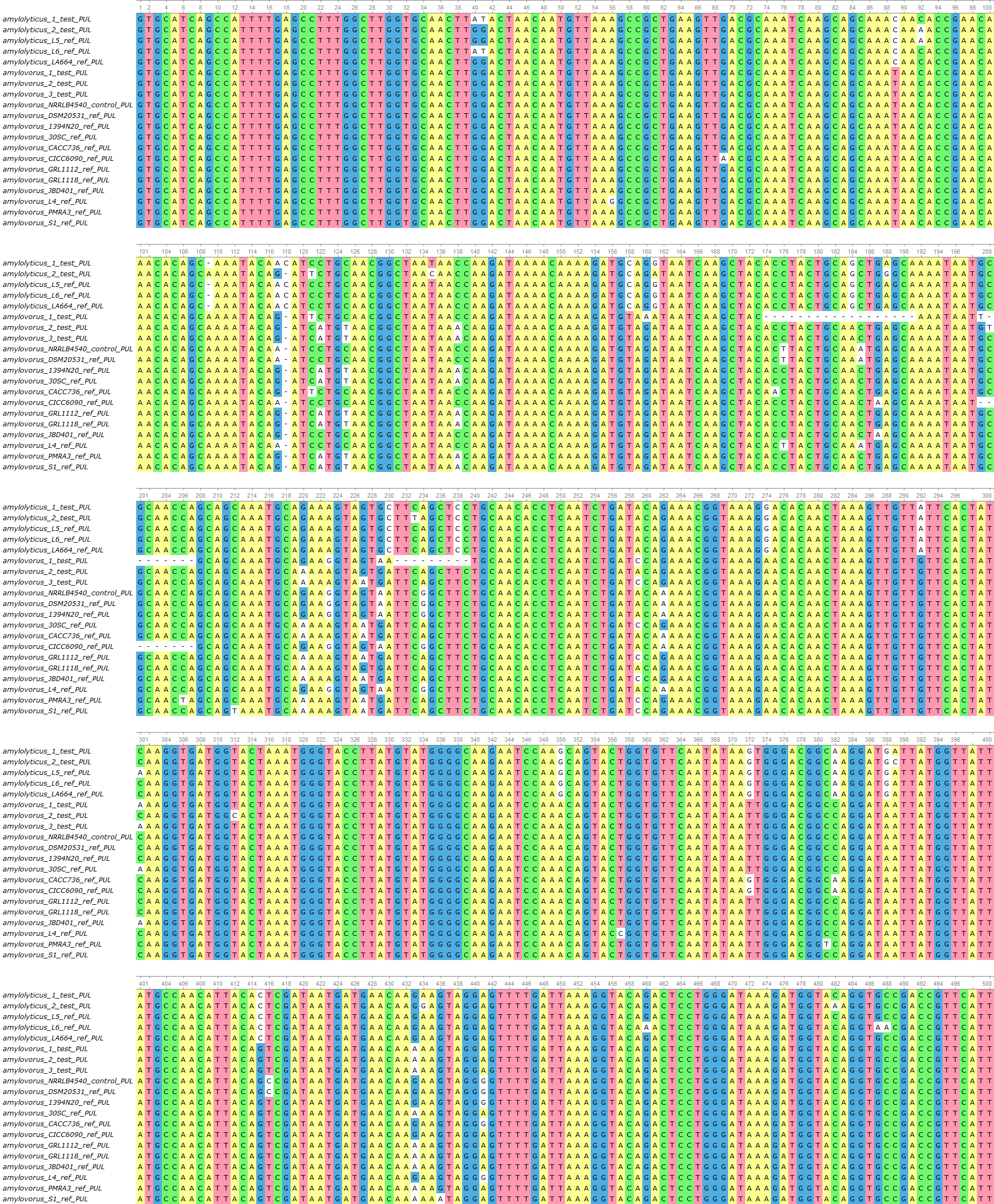


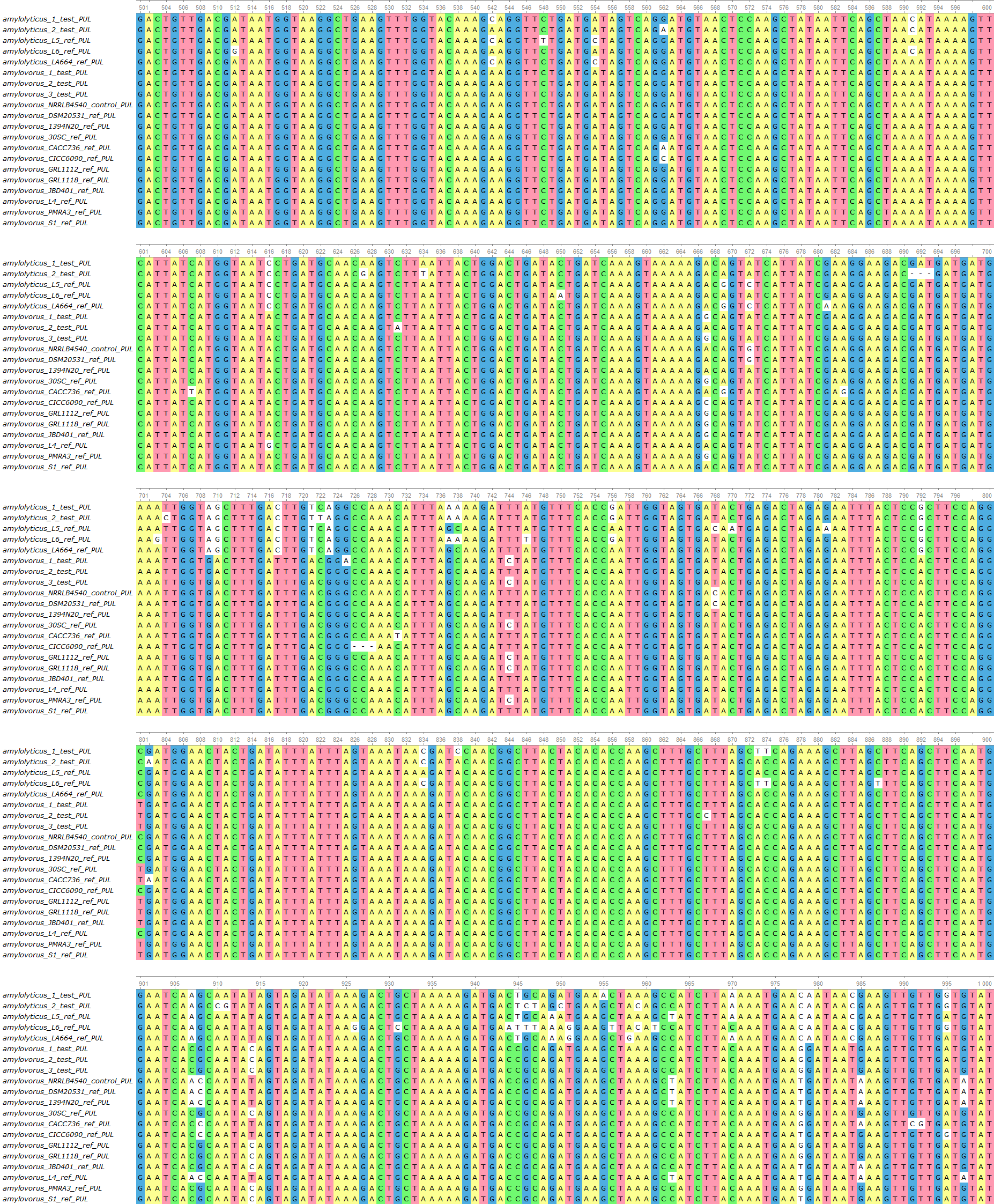


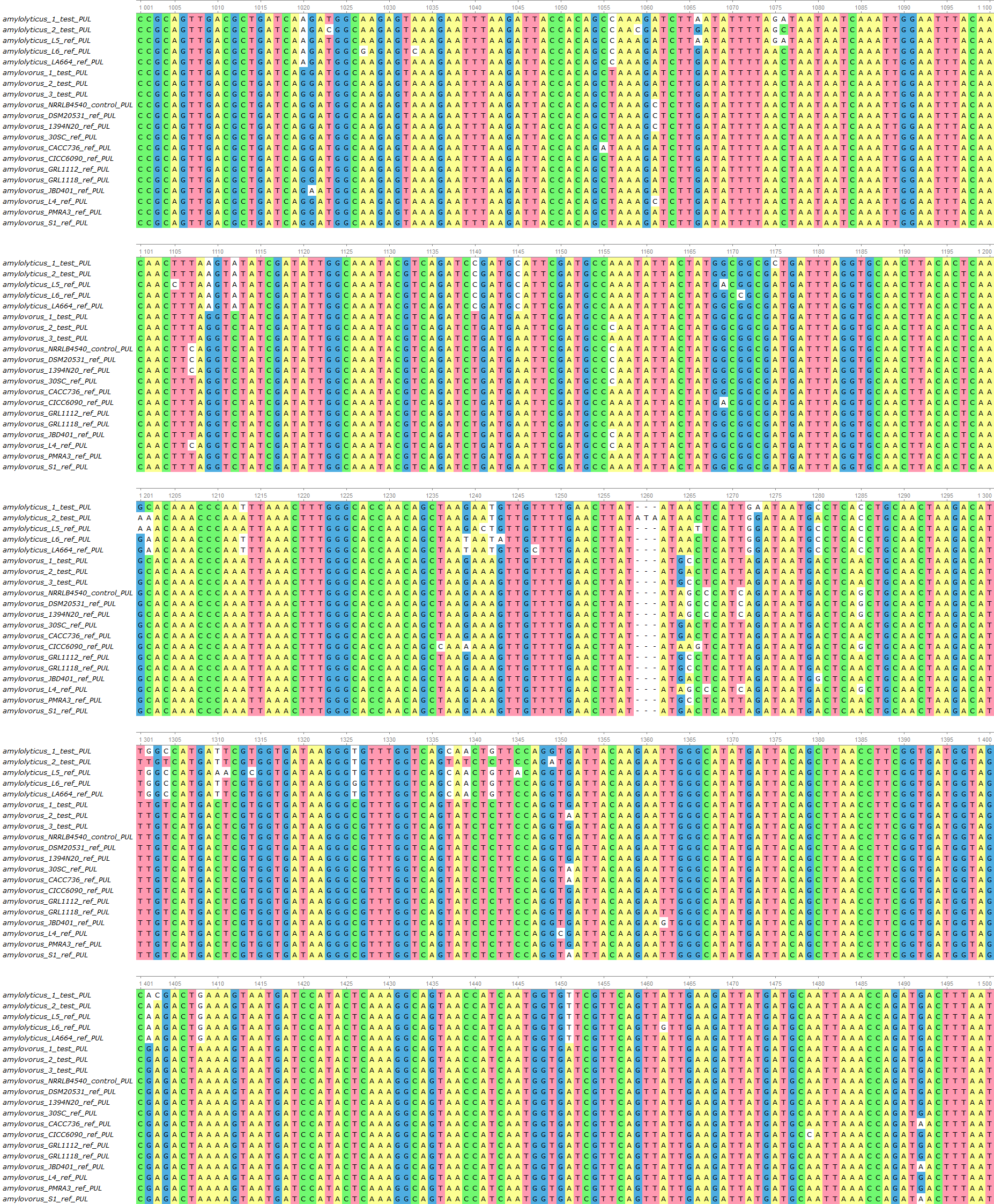


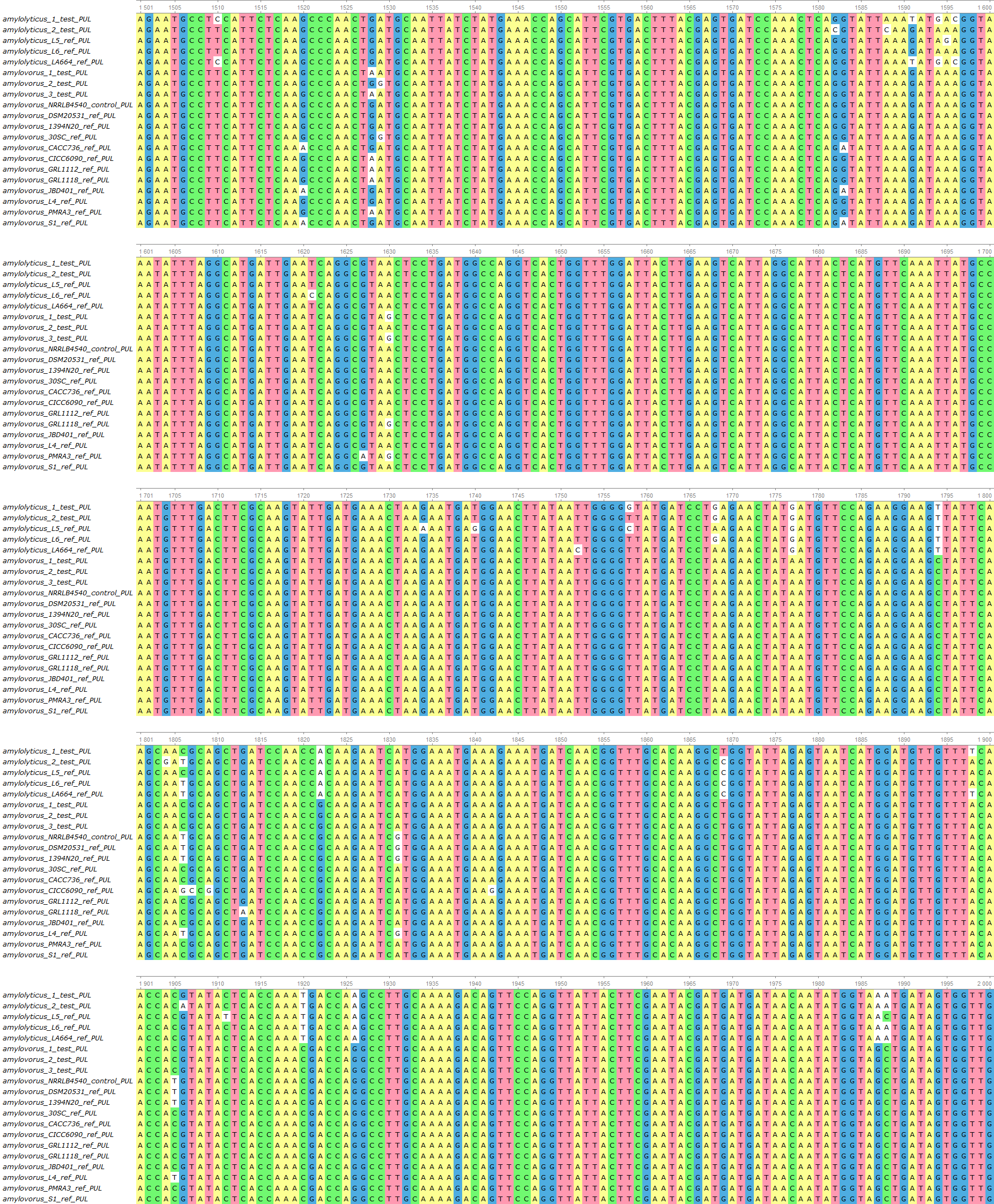


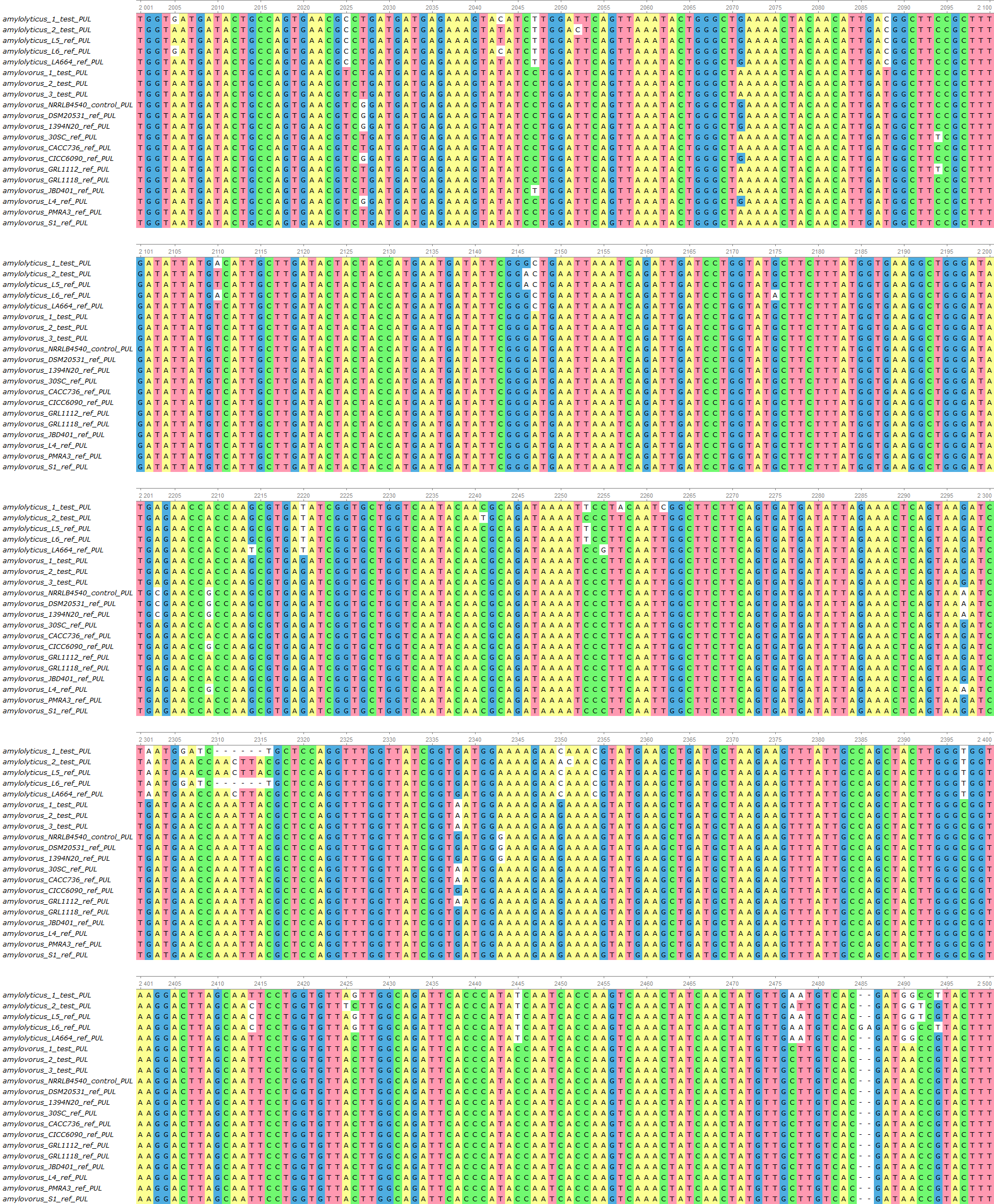


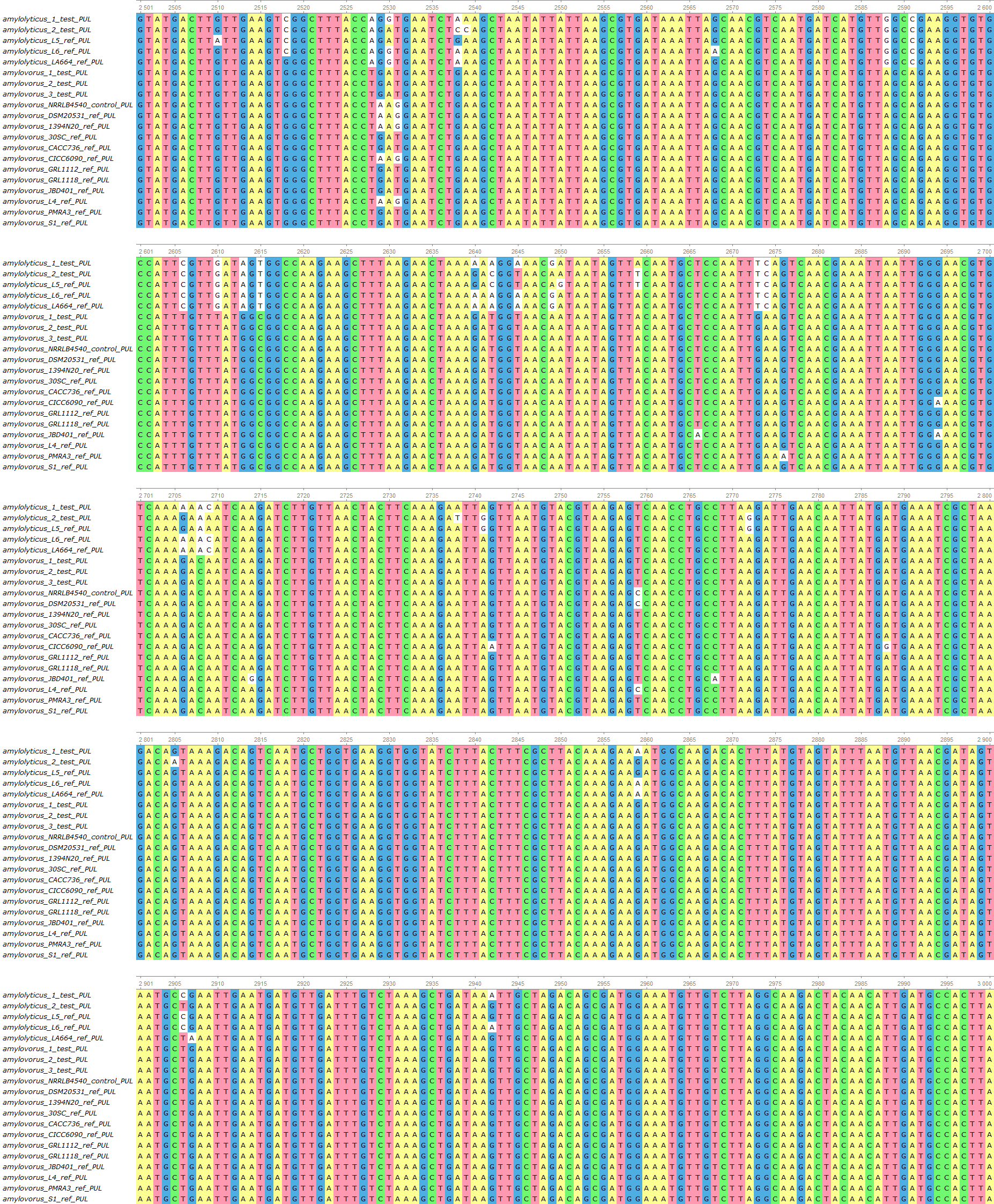


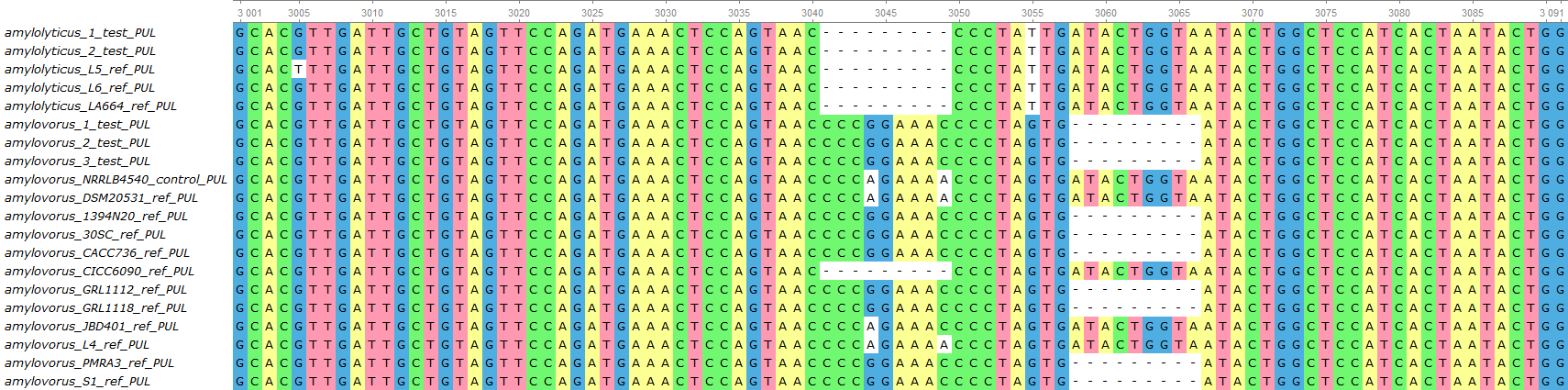


**Supplementary Figure 2.** MSA of *gly1* DNA sequences from test isolates and reference strains (trimmed).


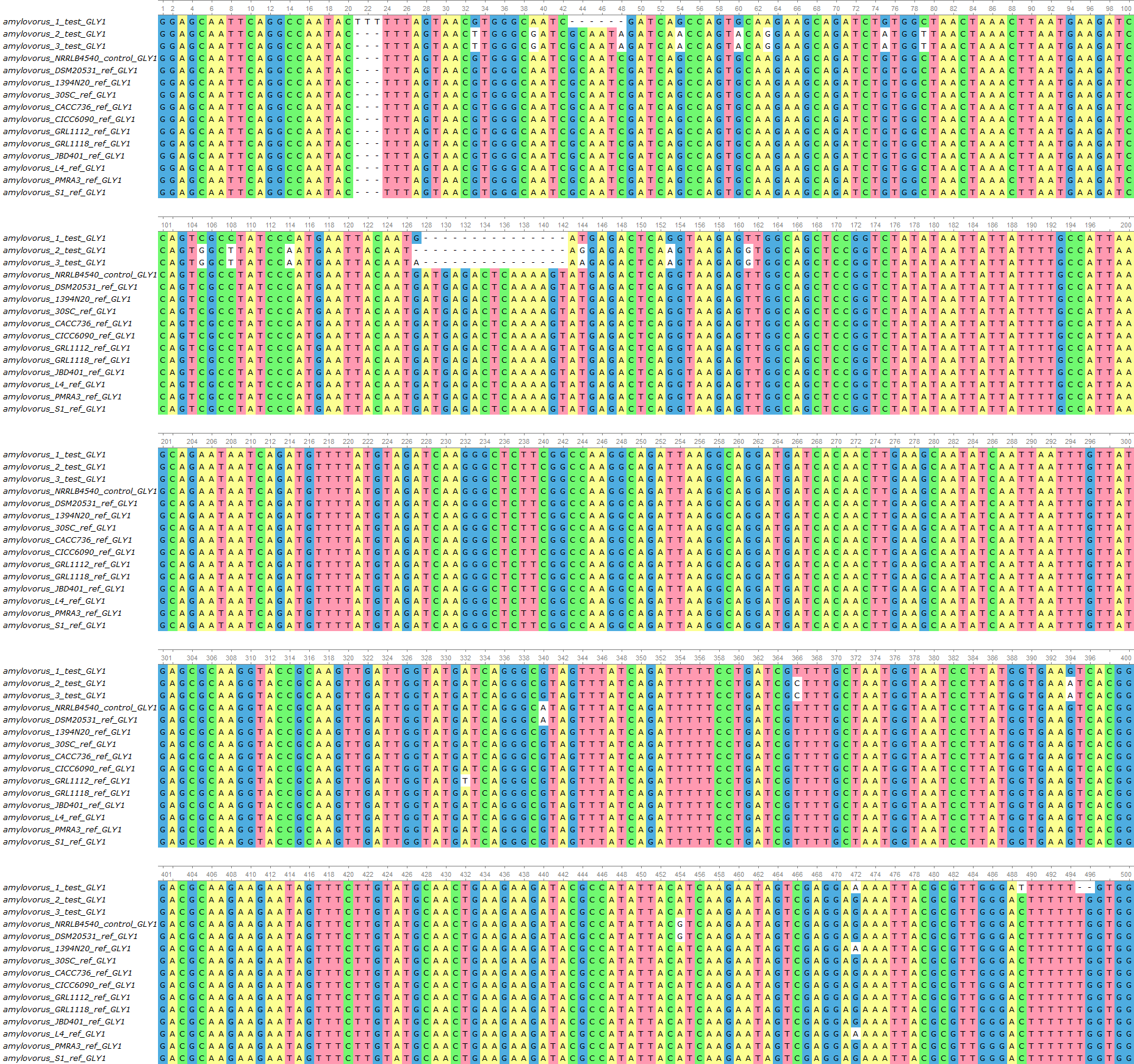


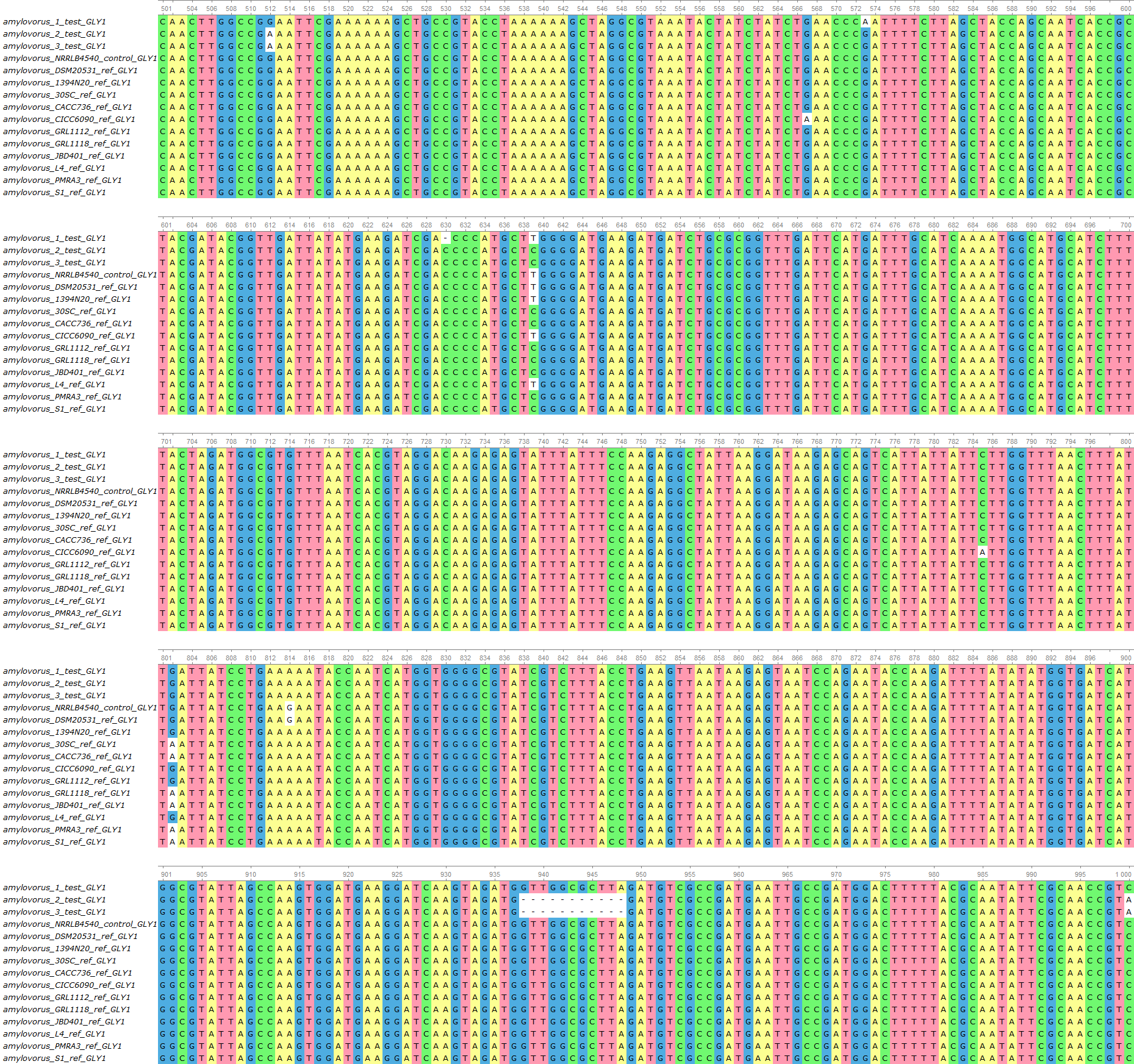


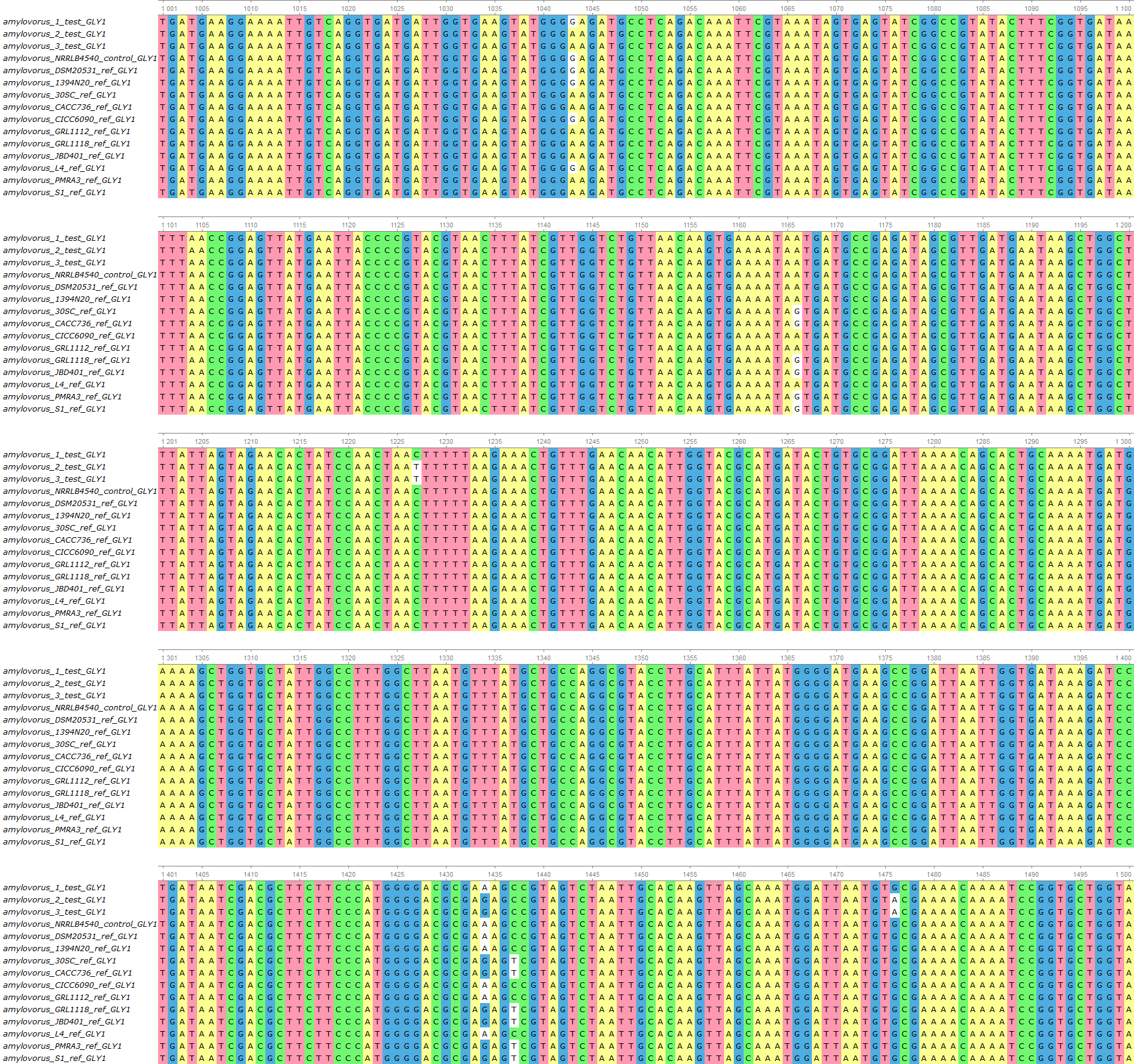


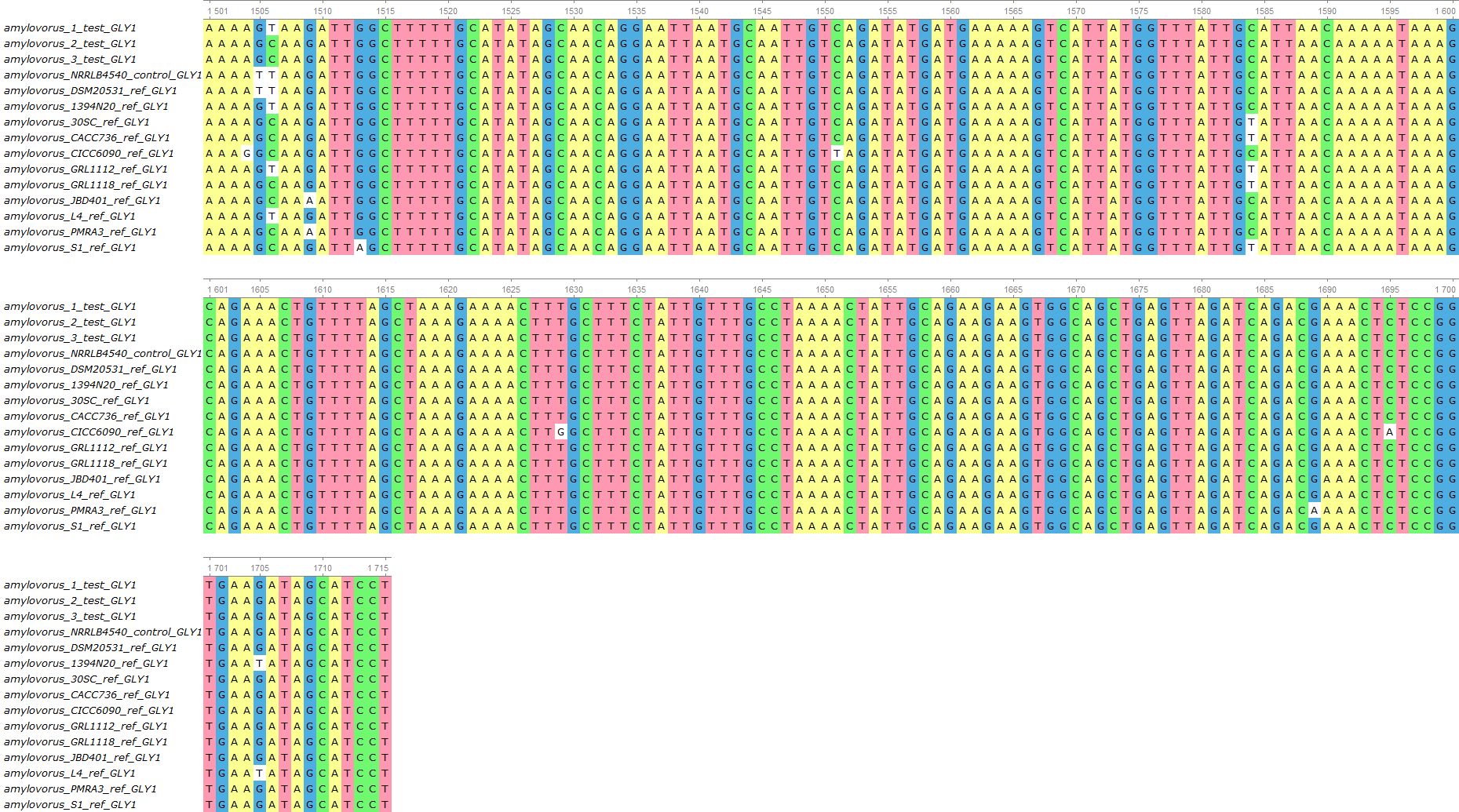


**Supplementary Figure 3.** MSA of *gly2* DNA sequences from test isolates and reference strains (trimmed).


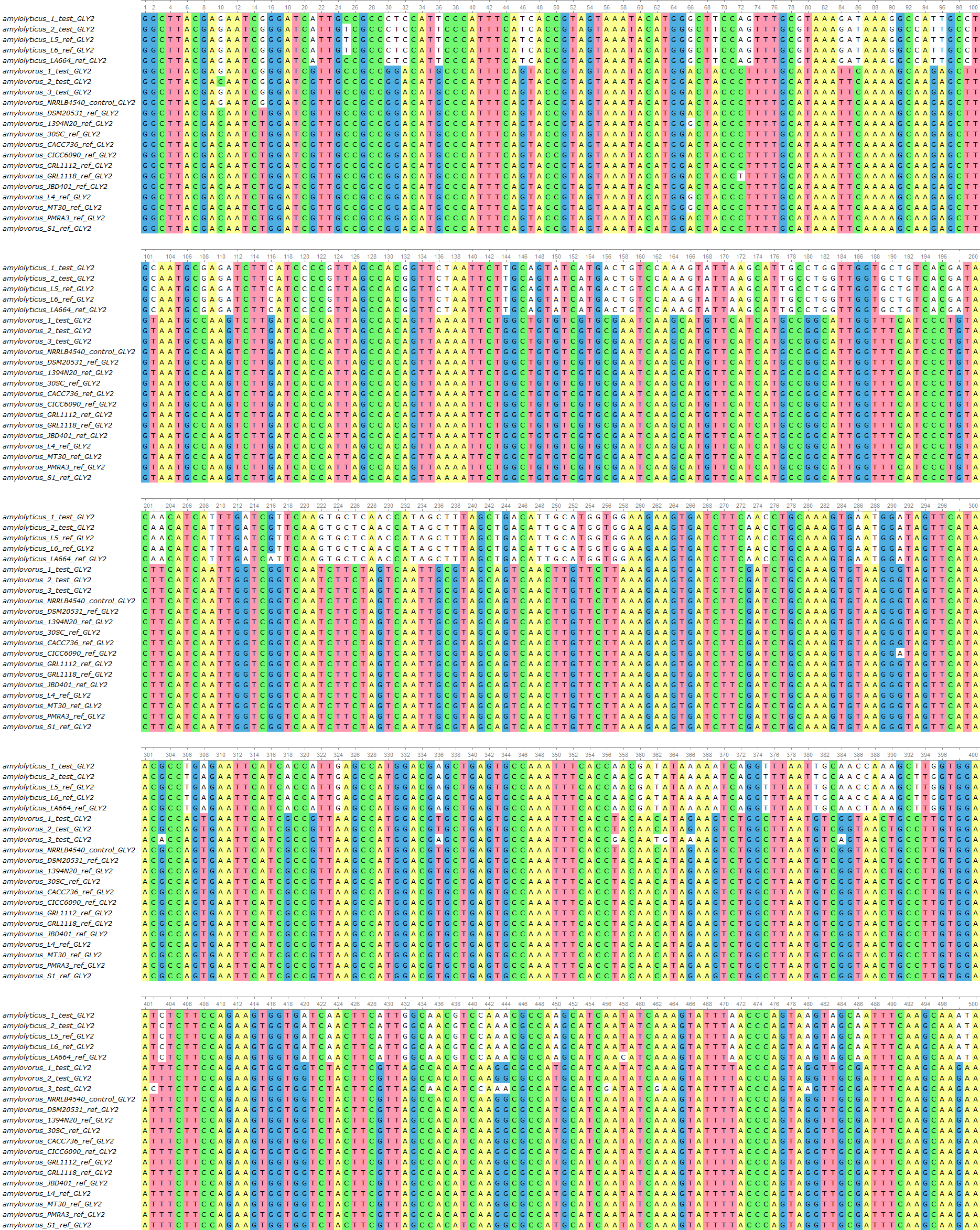


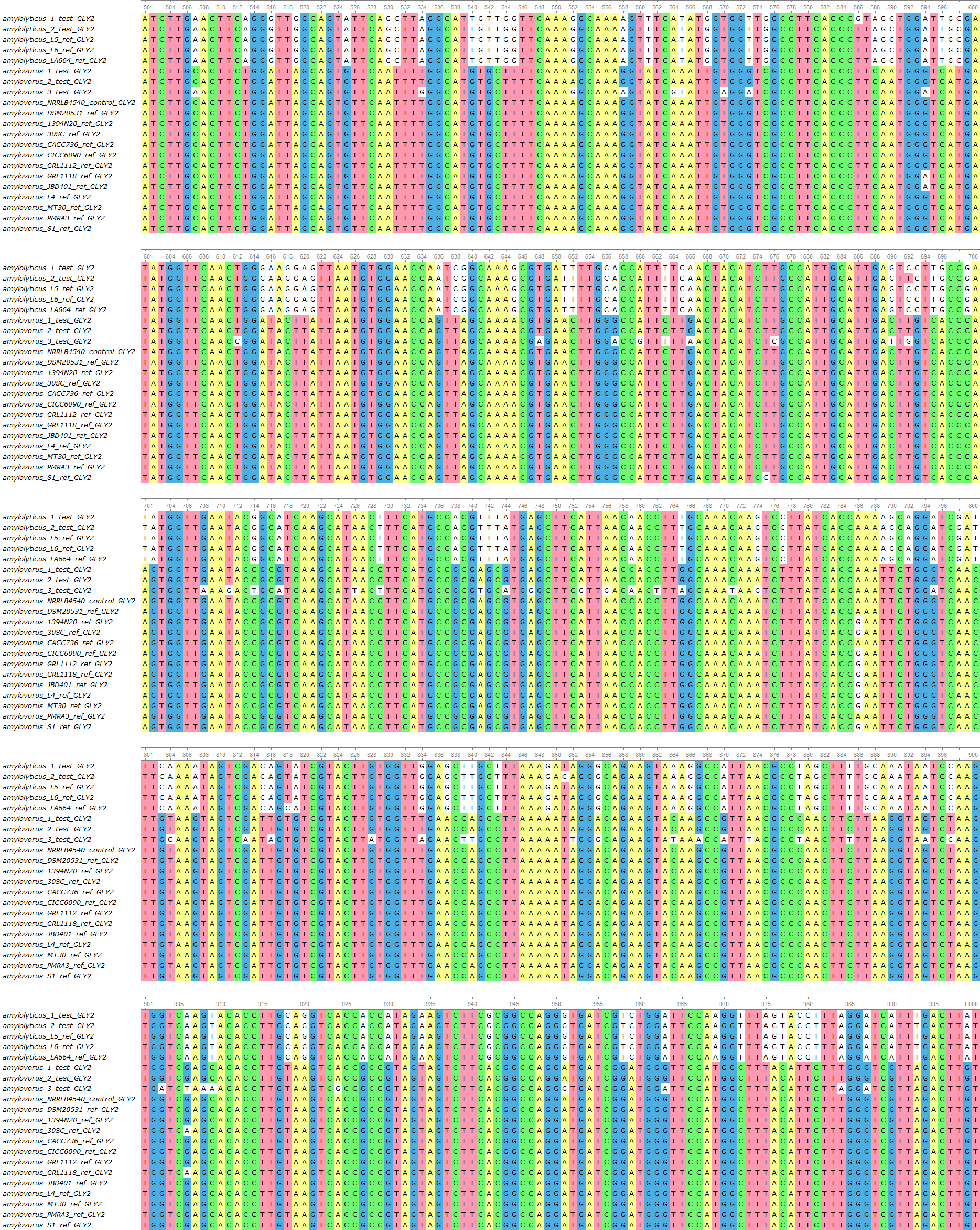


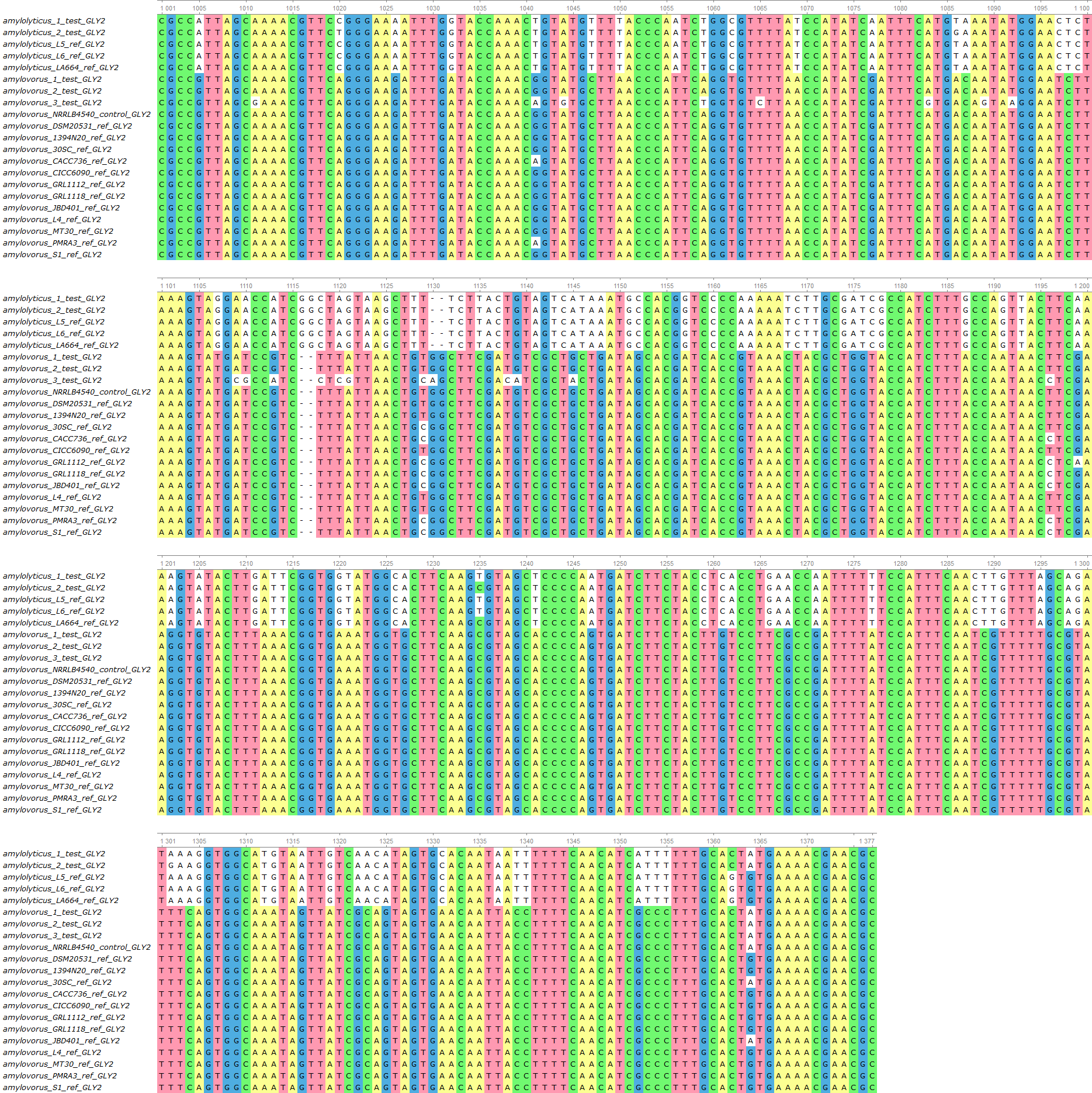


**Supplementary Figure 4.** MSA of Pul amino acid sequences from test isolates (deduced) and reference strains (trimmed).


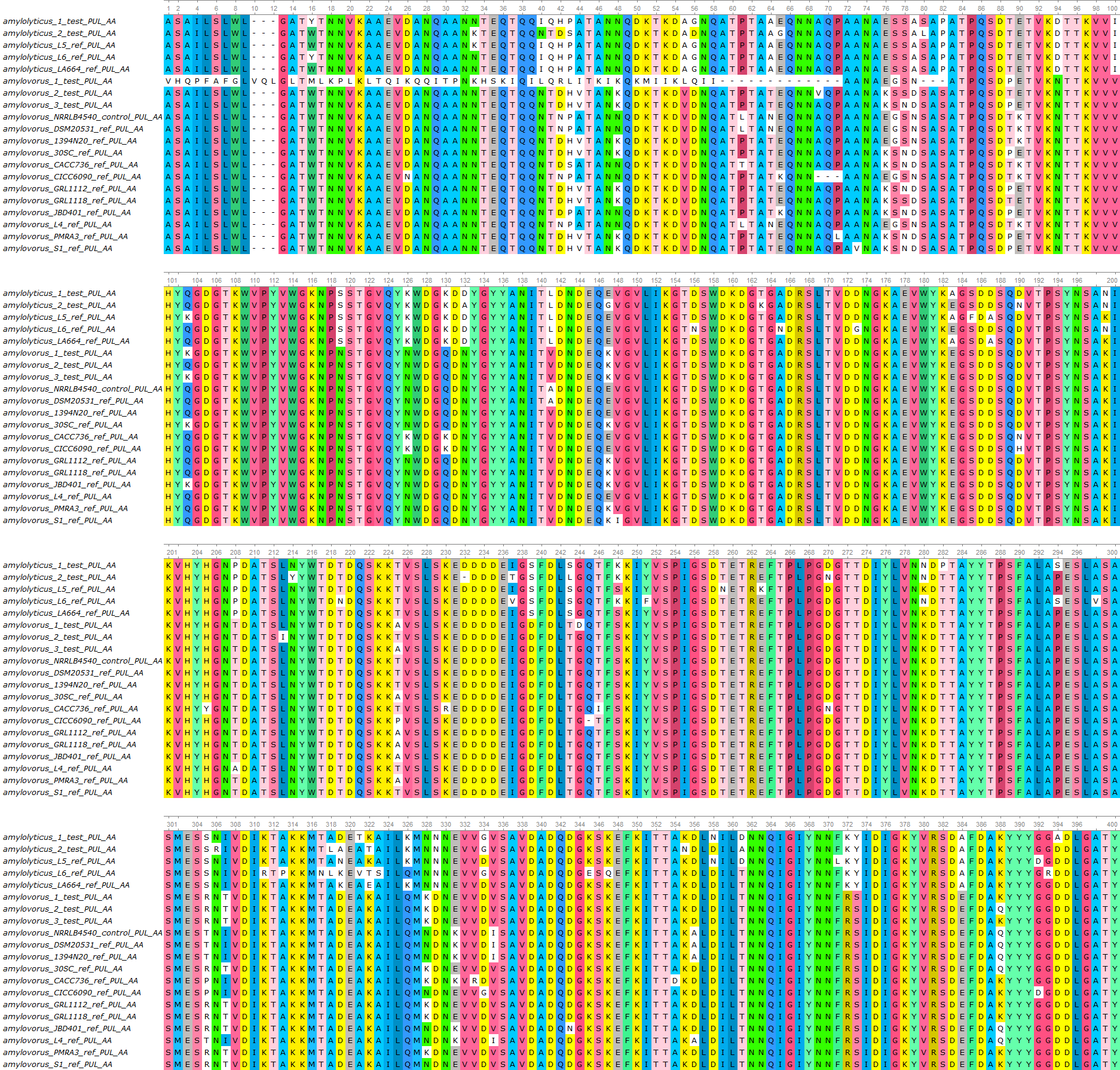


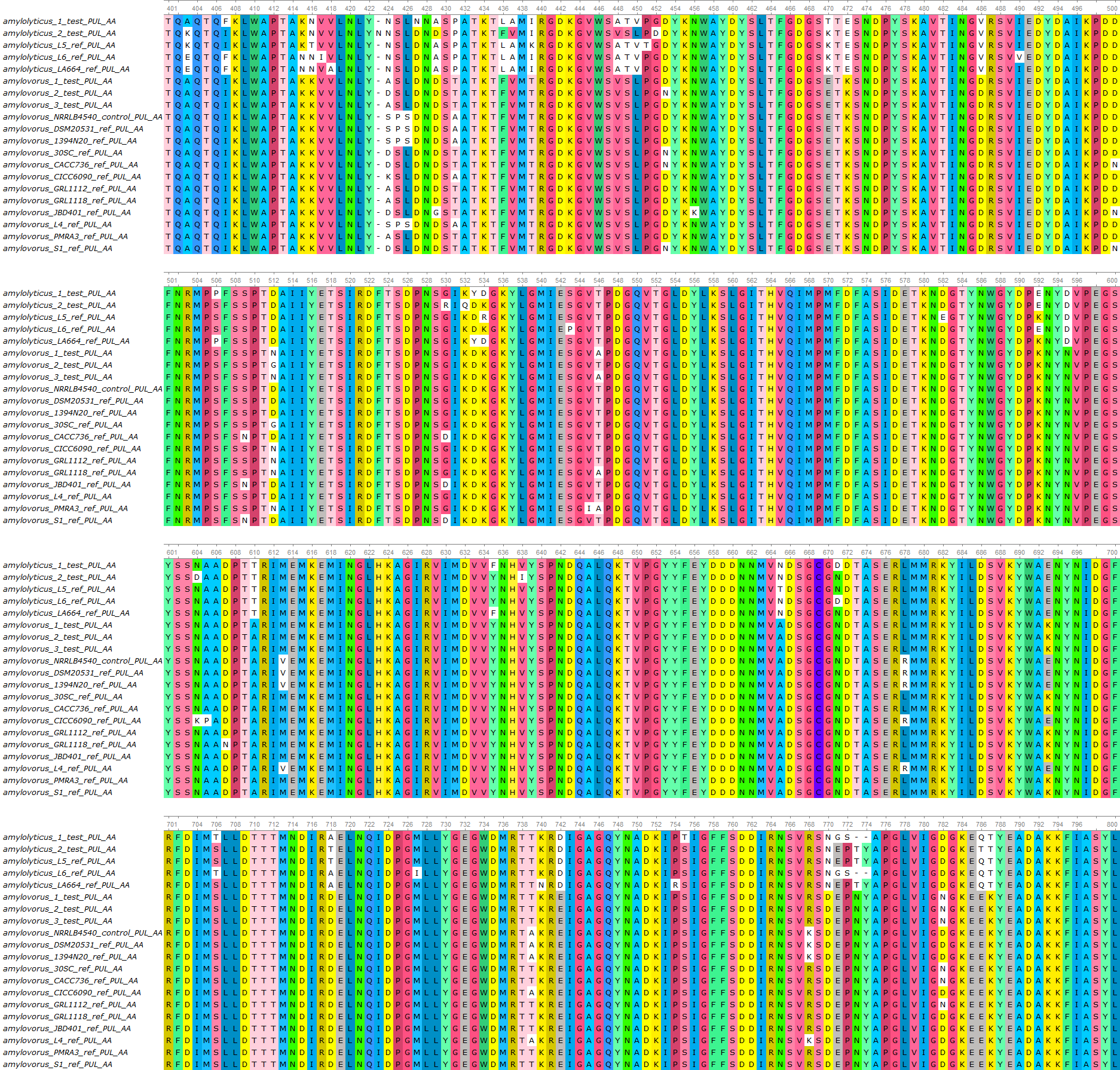


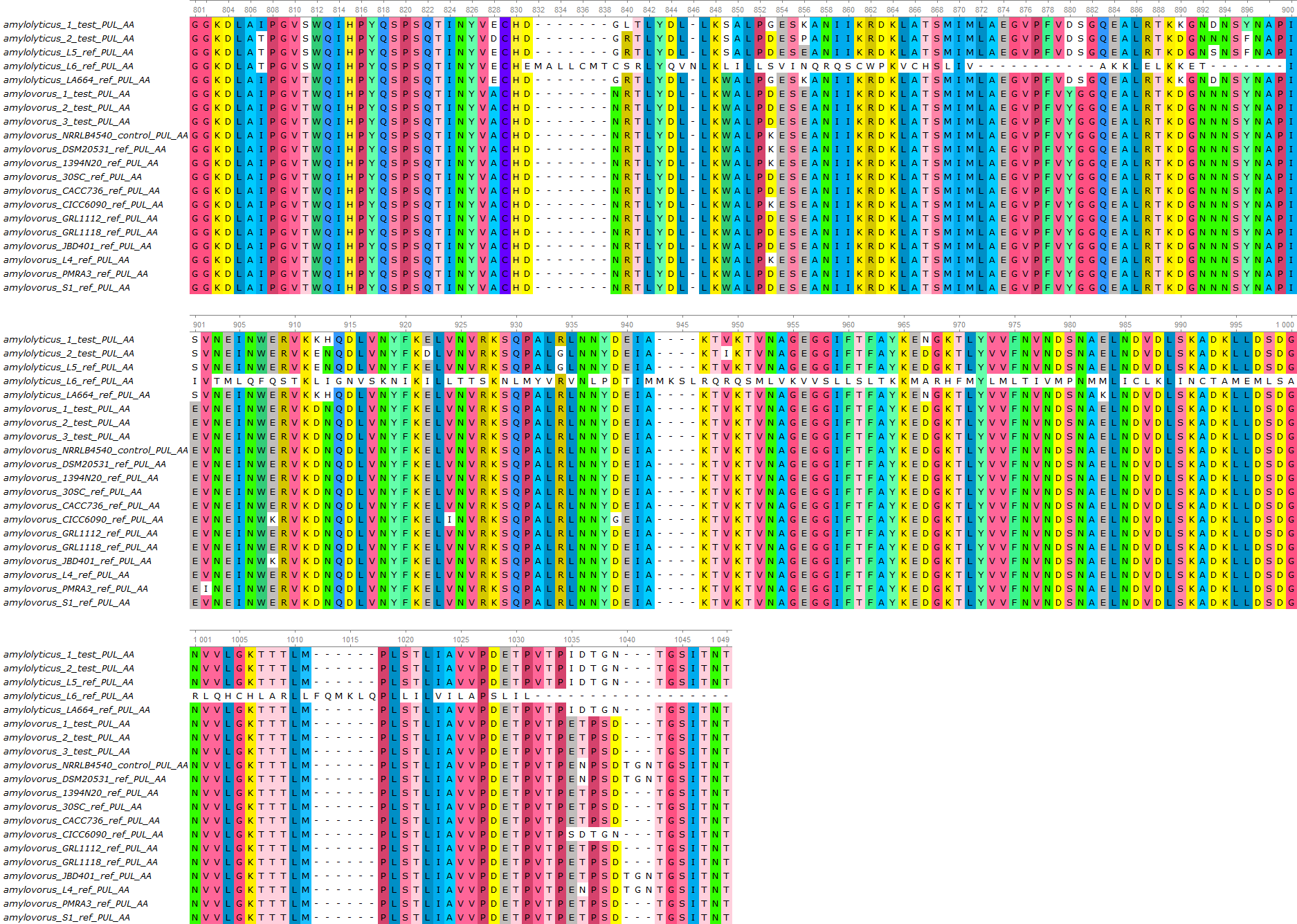


**Supplementary Figure 5.** MSA of Gly1 amino acid sequences from test isolates (deduced) and reference strains (trimmed).


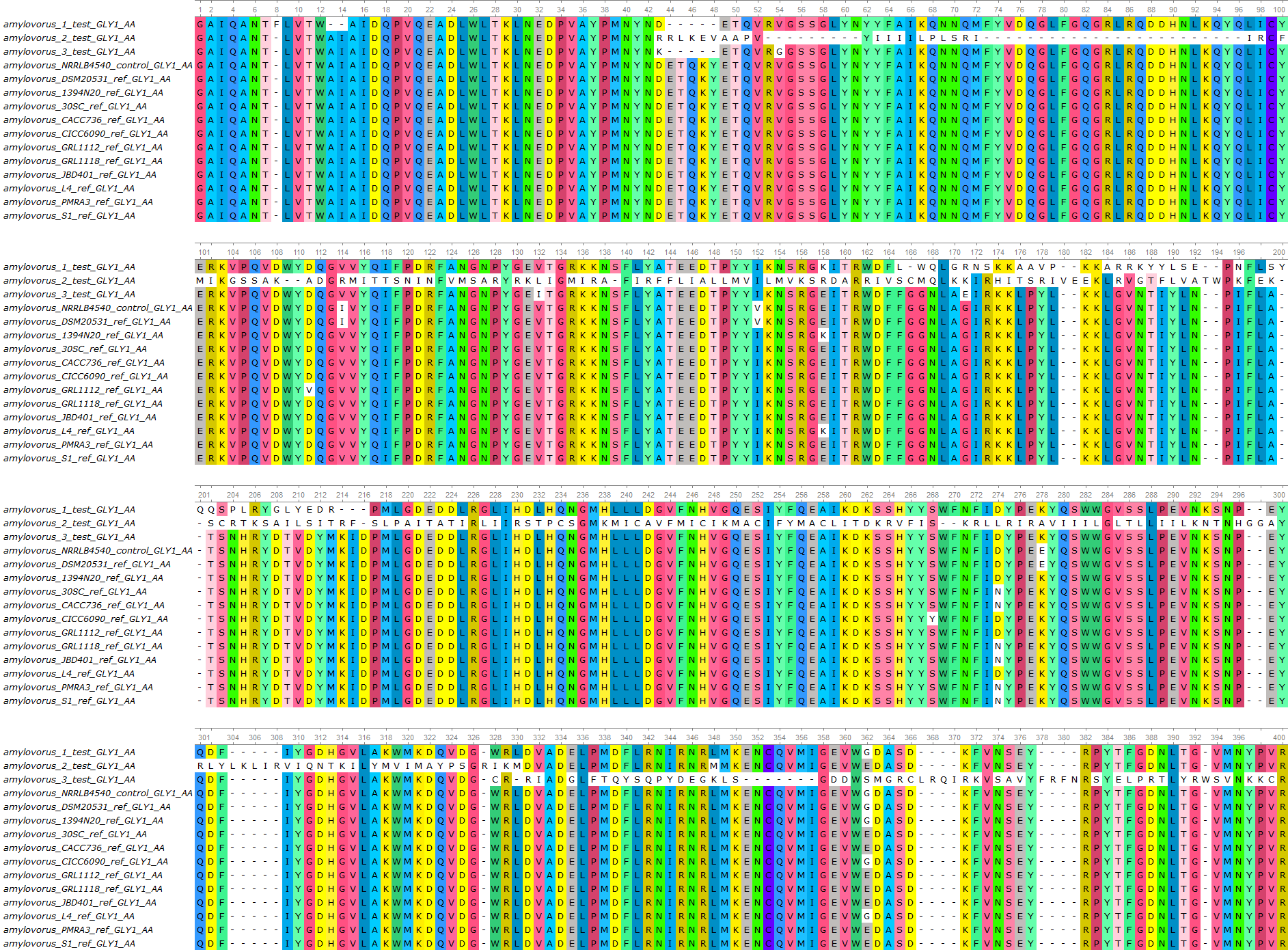


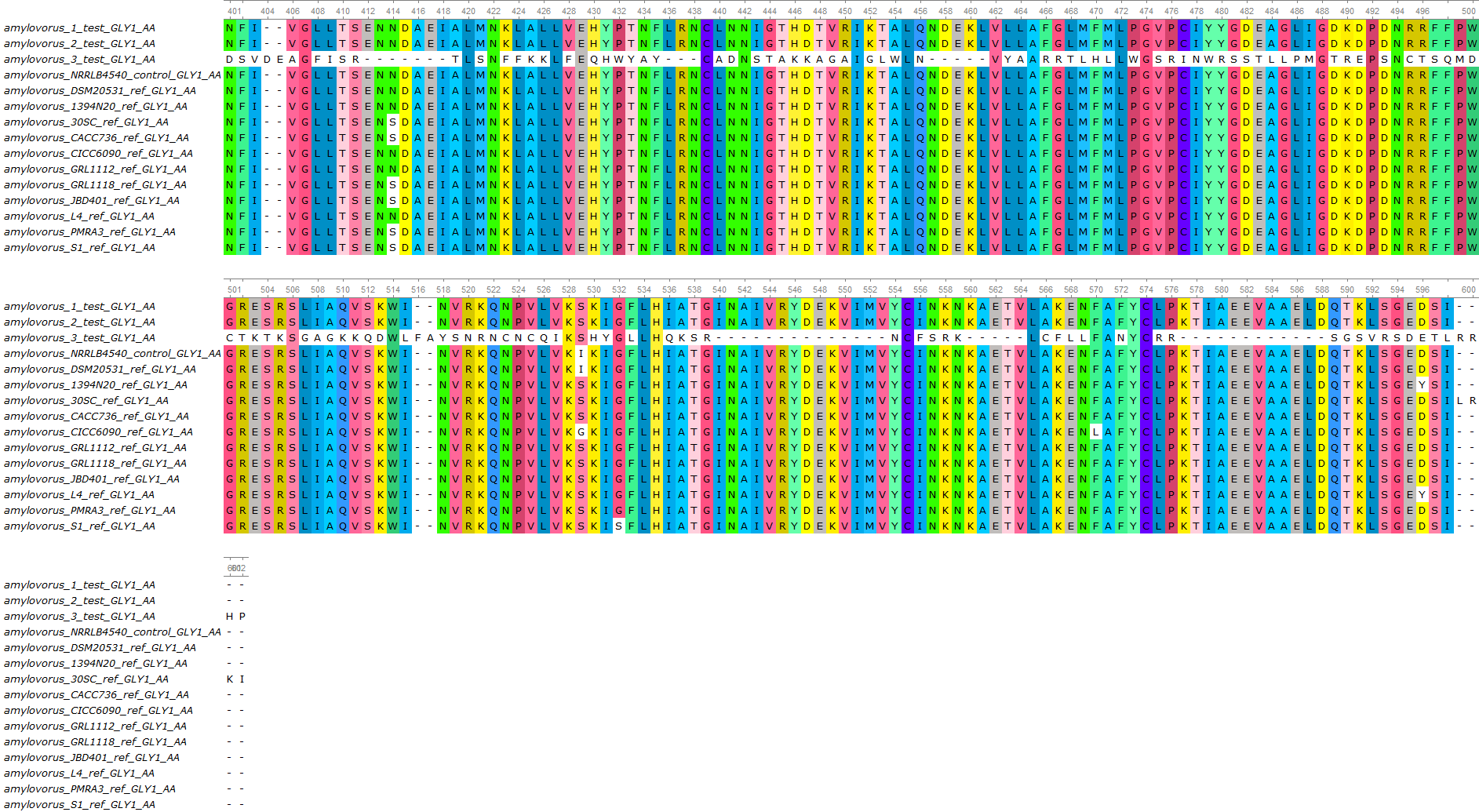


**Supplementary Figure 6.** MSA of Gly2 amino acid sequences from test isolates (deduced) and reference strains (trimmed).


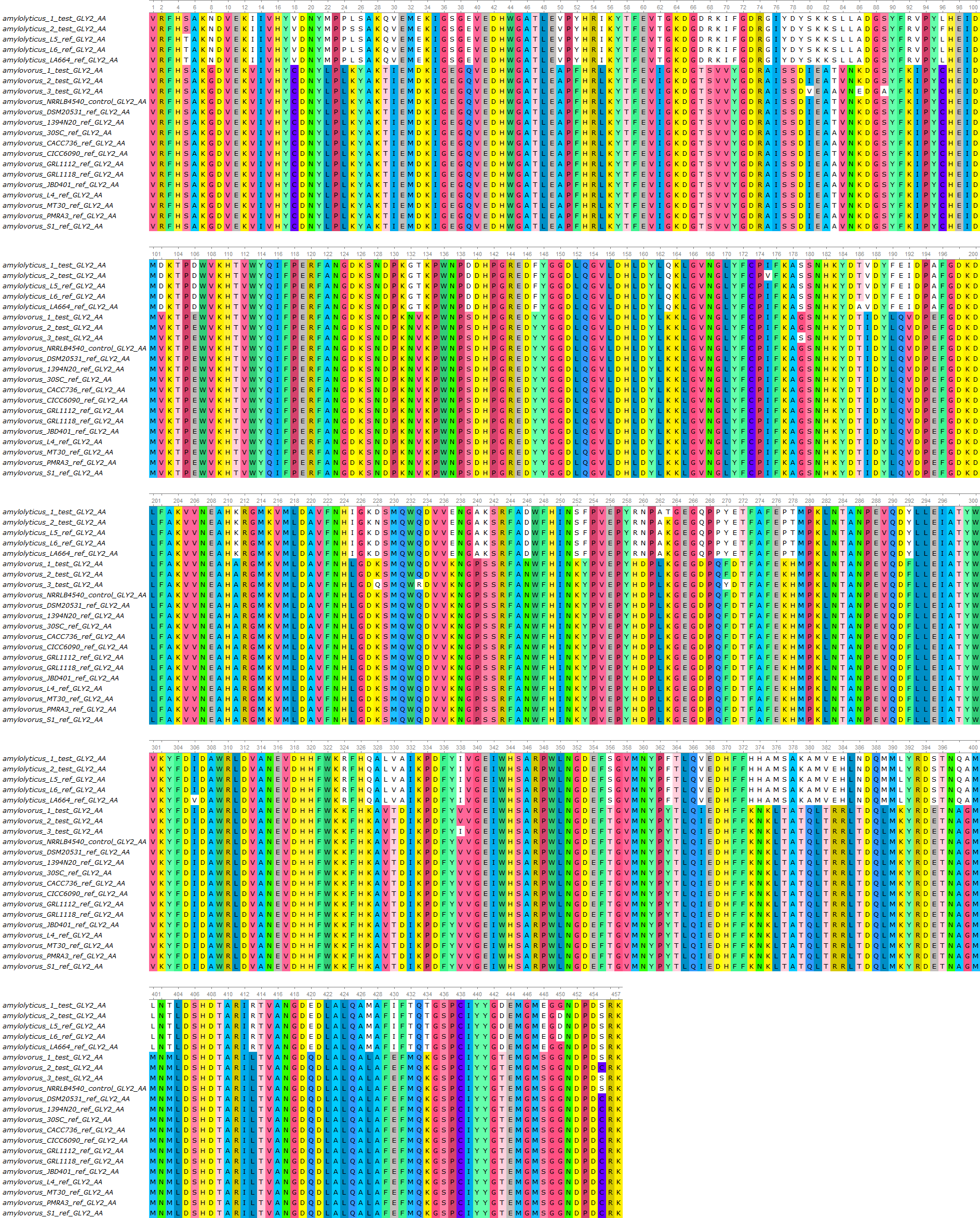

Supplement: Supplementary file 1 [file Data_Sheet_1.docx]
